# Rice OsMYB5P improves plant phosphate acquisition by regulation of phosphate transporter

**DOI:** 10.1371/journal.pone.0194628

**Published:** 2018-03-22

**Authors:** Won Tae Yang, Dongwon Baek, Dae-Jin Yun, Kwang Sik Lee, So Yeon Hong, Ki Deuk Bae, Young Soo Chung, Yong Sham Kwon, Du Hyun Kim, Ki Hong Jung, Doh Hoon Kim

**Affiliations:** 1 College of Life Science and Natural Resources, Dong-A University, Busan, Korea; 2 Division of Applied Life Science (BK21 PLUS), Plant Molecular Biology and Biotechnology Research Center, Gyeongsang National University, Jinju, Korea; 3 Department of Biomedical Science and Engineering, Konkuk University, Seoul, Korea; 4 Graduate School of Biotechnology and Crop Biotech Institute, Kyung Hee University, Yongin, Korea; National Taiwan University, TAIWAN

## Abstract

Myeloblastosis (MYB) transcription factors play central roles in plant developmental processes and in responses to nutrient deficiency. In this study, OsMYB5P, an R2R3-MYB transcription factor, was isolated and identified from rice (*Oryza sativa* L. ‘Dongjin’) under inorganic phosphate (Pi)-deficient conditions. OsMYB5P protein is localized to the nucleus and functions as a transcription activator in plant development. Overexpression of *OsMYB5P* in rice and *Arabidopsis* (*Arabidopsis thaliana* Col-0) increases tolerance to phosphate starvation, whereas *OsMYB5P* knock-out through RNA interference increases sensitivity to Pi depletion in rice. Furthermore, shoots and roots of transgenic rice plants overexpressing *OsMYB5P* were longer than those of wild plants under both normal and Pi-deficient conditions. These results indicate that *OsMYB5P* is associated with the regulation of shoot development and root- system architecture. Overexpression of *OsMYB5P* led to increased Pi accumulation in shoots and roots. Interestingly, OsMYB5P directly bound to MBS (MYB binding site) motifs on the *OsPT5* promoter and induced transcription of *OsPT5* in rice. In addition, overexpression of *OsMYB5P* in *Arabidopsis* triggered increased expression of *AtPht1;3*, an *Arabidopsis* Pi transporter, in shoots and roots under normal and Pi-deficient conditions. Together, these results demonstrate that overexpression of *OsMYB5P* increases tolerance to Pi deficiency in plants by modulating Pi transporters at the transcriptional level in monocots and dicots.

## Introduction

Inorganic phosphate (Pi) in plants is an important nutrient for growth and productivity [1, 2]. Pi influences the regulation of biological (*e*.*g*., energy metabolism, signal transduction, and enzyme regulation) and physiological processes (*e*.*g*., anthocyanin accumulation, and release of organic acids into the rhizosphere) in plants [2–6]. To maintain cellular Pi homeostasis under Pi-deficient conditions, plants modify their root-system architecture (RSA) by developing more lateral roots and root hairs [7–9]. These responses are achieved primarily by coordination of Pi acquisition from the soil, Pi translocation from roots to shoots, and internal Pi remobilization [10].

Pi uptake is mediated by plasma-membrane-localized Pi transporters (PTs), which are encoded by nine isolated genes (*PHT1;1*-*PHT1;9*) in *Arabidopsis* and 13 genes (*OsPT1*-*OsPT13*) in rice (*Oryza sativa*) [4, 11, 12]. These PTs, encoded by *PHT1* family members, are temporally and spatially upregulated in response to Pi starvation [13–15]. In *Arabidopsis*, the functions of most *PTs* under Pi starvation responses have been well elucidated, and the function of nine *OsPTs* in rice have also been characterized [11, 16–18]. Among these *OsPTs*, *OsPT1* is highly expressed in roots and is a major regulator of Pi acquisition to maintain Pi homeostasis [19]. *OsPT6*, *OsPT9*, and *OsPT10* are highly expressed in roots during low Pi stress and are associated with Pi uptake and translocation [20, 21]. *OsPT2*, a low-affinity transporter, plays an important role in Pi translocation from the root to the shoot, whereas *OsPT8*, a high-affinity transporter, plays a role in Pi uptake capacity in Pi homeostasis responses [22, 23]. *OsPT4* transcripts are expressed constitutively in shoots and roots under Pi-deficient conditions [24]. In addition, *OsPT11* and *OsPT13*, exclusively expressed in roots, are involved in fungal (such as arbuscular mycorrhizal) symbioses and symbiotic Pi uptake [16, 25]. Therefore, in response to plant Pi starvation, PT activity is increased accordingly in plant tissues [4, 12].

Although many studies have reported Pi-related transcription factors, the functions of several transcription factors that are directly regulated phosphate transporters in Pi starvation still have not been characterized in rice. For example, transcription factors such as PHR1 [26], PHL1/2/3 [27, 28], WRKY75 [29], WRKY45 [30], WRKY42 [31], and OsPHR1/2/3 [32, 33] have been reported to regulate *Arabidopsis* or rice *PHT1;1* expression under Pi-deficient or Pi-sufficient conditions. In particular, *Arabidopsis* PHR1 binds to the P1BS (PHR1-binding sequence) motif as a dimer to a palindromic sequence (*GNATATNC*). AtPHR1 orthologs, such as OsPHR2 (*Oryza sativa*) [32], PvPHR1 (*Phaseolus vulgaris*) [34], BnPHR1 (*Brassica napus*) [35] and TaPHR1 (*Triticum aestivum*) [36], have been reported as having similar functions in Pi signaling and homeostasis.

Despite the research that has been done in this area, the molecular mechanism whereby R2R3-type MYB transcription factors directly regulate expression of *PTs* in rice is not as well known, except for OsMYB2P-1 and OsMYB4P [37, 38]. In this study, we investigated the roles of rice MYB5P in modulating Pi homeostasis through regulating *PTs* expression in rice and *Arabidopsis*. In particular, we found that OsMYB5P modulates Pi uptake by directly regulating *OsPT5* expression under Pi-deficient and Pi-sufficient conditions in rice. OsMYB5P was found to be localized to the nucleus and to function as a transcriptional activator. Moreover, Pi acquisition was significantly increased by overexpression of *OsMYB5P* in rice and *Arabidopsis*. We demonstrated, by using a combination of physiological and biological approaches, that OsMYB5P, which functions as a transcriptional activator essential for *PTs* expression, plays an important role in maintaining Pi homeostasis in plants. Understanding the mechanism undergirding the maintenance of Pi homeostasis by *OsMYB5P* in monocots and dicots will help to develop new cultivars with high Pi efficiency.

## Materials and methods

### Plant materials and growth conditions

*Oryza sativa* L. ‘Dongjin’ and *Arabidopsis thaliana* Col-0 plants were used in all physiological experiments and were also used to generate transgenic plants. We performed hydroponic and suspension cell-culture experiments as described in detail previously [38]. Rice or *Arabidopsis* plants were cultivated in growth chambers at 32 °C or 22 °C.

### *In silico* analysis

To conduct an *in silico* analysis of *OsMYB5P* (*Os02g0624300*), sequences were analyzed by the BLAST sequence and multiple sequence alignment (MSA) programs from NCBI (http://blast.ncbi.nlm.nih.gov/Blast.cgi), Gramene (http://www.gramene.org), and CLUSTAL W (http://www.genome.jp/tools/clustalw).

### Plasmid construction

To construct OsMYB5P-OX and OsMYB5P-RNAi transgenic rice plants, we inserted the full length (774 bp) and a partial fragment (210 bp) of the *OsMYB5P* gene into *pENTR*^*™*^/*D-TOPO* (Invitrogen, Carlsbad, CA, USA). All gene-specific primer sequences are listed in S3 Table. The recombination reaction between the entry and destination vectors was carried out using LR Clonase^™^ II enzyme mix (Invitrogen, Carlsbad, CA, USA) according to the manufacturer’s instructions. The destination vectors used were *pH7WG2D*.*1* and *pB7GWIWG2(II)*,*0*.

### Generation of OsMYB5P transgenic plants

The *OsMYB5P*:*pH7WG2D*.*1* (OsMYB5P-OX) and *OsMYB5P*:*pB7GWIWG2(II)*,*0* (OsMYB5P-RNAi) constructs were introduced into *Agrobacterium tumefaciens* (EHA105) by electroporation. We used a modified version of a general rice-transformation protocol [39, 40]. Rice seeds were placed on N6D callus induction medium. Callus growth was induced by culturing at 30 °C in the dark for 4 weeks. Actively growing embryogenic calli were used in this experiment. Calli induced on N6D media were transferred to fresh media and preconditioned for 3 d at 28 °C in the dark. Preconditioned calli as described above were immersed in *A*. *tumefaciens* suspension for 10 min and transferred to 2N6-AS medium. To improve co-cultivation efficiency, 100 μM of acetosyringone was added to the bacterial suspension after 1 d of culture. After the co-cultivation, calli were rinsed two times with distilled water, three times with distilled water containing 200 mg/L cefotaxime, and placed on the first selection media. Calli were cultured at 26 °C for 2 weeks. After 2 weeks, rapidly growing calli that had proliferated on the selection media were transferred to regeneration media biweekly until roots and shoots emerged. Then, transgenic OsMYB5P-OX (T0) plants were transferred to soil and were grown in growth chambers at 32 °C.

*Agrobacterium tumefaciens* (*GV3101*)-mediated *Arabidopsis* transformation was performed using vacuum infiltration [41]. Transgenic OsMYB5P-OX (T1) plants were selected on Murashige and Skoog media containing the appropriate antibiotics (Kanamycin) and then transferred to soil and allowed to self-pollinate.

### Transient analysis in *Arabidopsis* protoplasts

To investigate the subcellular localization and transcriptional activity of OsMYB5P, we introduced plasmid constructs into *Arabidopsis* protoplasts prepared from leaf tissues by PEG-mediated transformation, as described in detail previously [38, 42].

### Gene expression analysis

As previously reported, total RNA was isolated using the RNeasy Kit (Qiagen, Valencia, CA, USA) according to the manufacturer’s instructions for northern blot and quantitative real-time PCR (qRT-PCR) analysis [38]. First-strand cDNAs were synthesized using 3 μg of total RNA with a cDNA Synthesis Kit (Invitrogen, Carlsbad, CA, USA), to serve as the templates for qRT-PCR. To measure levels of gene expression, qRT-PCR was performed, and values were automatically calculated using a CFX384 Real-time PCR Detection System and CFX Manager software (Bio-Rad. Hercules, CA, USA) following a standard protocol. The sequences of primers used in qRT-PCR analysis are provided in S3 Table.

### Measurements of total Pi content in plants

Samples were frozen after fresh weight measurement, or dried at 80 °C for 3 d to measure dry weight. Inorganic Pi measurement followed a previously described method [38].

### Protein expression and purification

We inserted the full-length *OSMYB5P* cDNA from rice into the *pGEX-2T* vector (Amersham, Buckinghamshire, UK) using *Xho*I restriction sites at the 5′ and 3′ ends of the cDNA fragment (S3 Table). The *pGEX-2T*:*OsMYB5P* clone was introduced into the BL21 DE3 strain (Merck KGaA, Darmstadt, Germany) of *E*. *coli*. Expression of OSMYB5P protein was induced by applying 0.5 mM IPTG for 3 h at 30 °C. The recombinant OSMYB5P-GST protein extracts were purified by affinity chromatography using glutathione-agarose resin (Amersham, Buckinghamshire, UK) according to the manufacturer’s recommendations.

### Preparation of OsMYB5P antibody

Purified recombinant OsMYB5P (10 μg) was mixed with an equal volume of Freund’s complete adjuvant (Sigma Chemical Co., St. Louis, MO, USA), for a total volume of 200 μL, and injected into BALB/c mice (Semtaco Bio Korea Co., South Korea). After the first injection, three successive injections were given at one-week intervals with antigens mixed with equal volumes of Freund’s incomplete adjuvant (a total of 200 μL, Sigma). Blood was collected 3 d after the last injection and centrifuged at 13,000 rpm for 5 min.

### Chromatin immunoprecipitation (ChIP) assay

We performed ChIP assays as described by [43]. Nuclei were isolated from 10-day-old plants (100 mg) using CelLytic^™^ PN (Sigma, St. Louis, MO, USA). Chromatin was extracted from nuclei by sonication 10× for 30 s at low power using a BIORUPTOR (BMS, Tokyo, Japan). Anti-OsMYB5P was used for immunoprecipitation. The amount of immunoprecipitated DNA was quantified by qRT-PCR. The primers used in the ChIP assay are listed in S3 Table.

### Electrophoretic mobility shift assay (EMSA)

To generate the biotin-labeled DNA probes, oligonucleotides spanning the MYB-binding sites (MBS) on the PTs promoter were used with 3′ overhang biotin-labeled oligonucleotides (OsPT5-MBS1-F, 5′-*TATAATATAATGAACTTACAGTTGAGACATACATATGAA*-3′; OsPT5-MBS1-R, 5′-*TTCATATGTATGTCTCAACTGTAAGTTCATTATATTATA*-3′; OsPT5-MBS2-F, 5′-*GGACAGGAACATTCCAGTTGATGGTTTCCTTCAATTAGG*-3′; OsPT5-MBS2-R, 5′-*CCTAATTGAAGGAAACCATCAACTGGAATGTTCCTGTCC*-3′; COSMO Genetech, Seoul, Korea). Biotin-labeled DNA was detected using a LightShift Chemiluminescence EMSA Kit (Thermo Fisher Scientific, IL, USA) according to the manufacturer’s instructions. The DNA binding reaction was allowed to proceed at 25 °C for 20 min in binding buffer (100 mM Tris *p*H 7.5, 500 mM KCl, 10 mM dithiothreitol), 5 mM MgCl_2_, 2.5% glycerol, 0.05% NP-40, and 50 ng/μL of poly (dI-dC), and with various concentrations of purified bacterial expressed OsMYB5P protein. The reaction was initiated by adding a 3′-biotin-labeled DNA probe and allowed to proceed at 25 °C for 30 min. The reaction mixture was then subjected to electrophoresis on a 6% polyacrylamide gel in 0.5× TBE buffer at 100 V for 3 h. The gel was then transferred to a Hybrid-N^+^ membrane (GE Healthcare, Buckinghamshire, UK), and cross-linked using a commercial UV-light crosslinking instrument (Stratagene/HiTechTrader, NJ, USA). The signal was automatically developed and exposed using a ChemiDoc^™^ image system (Bio-Rad, Hercules, CA, USA).

## Results

### Characterization of OsMYB5P in rice

**Fig 1:**
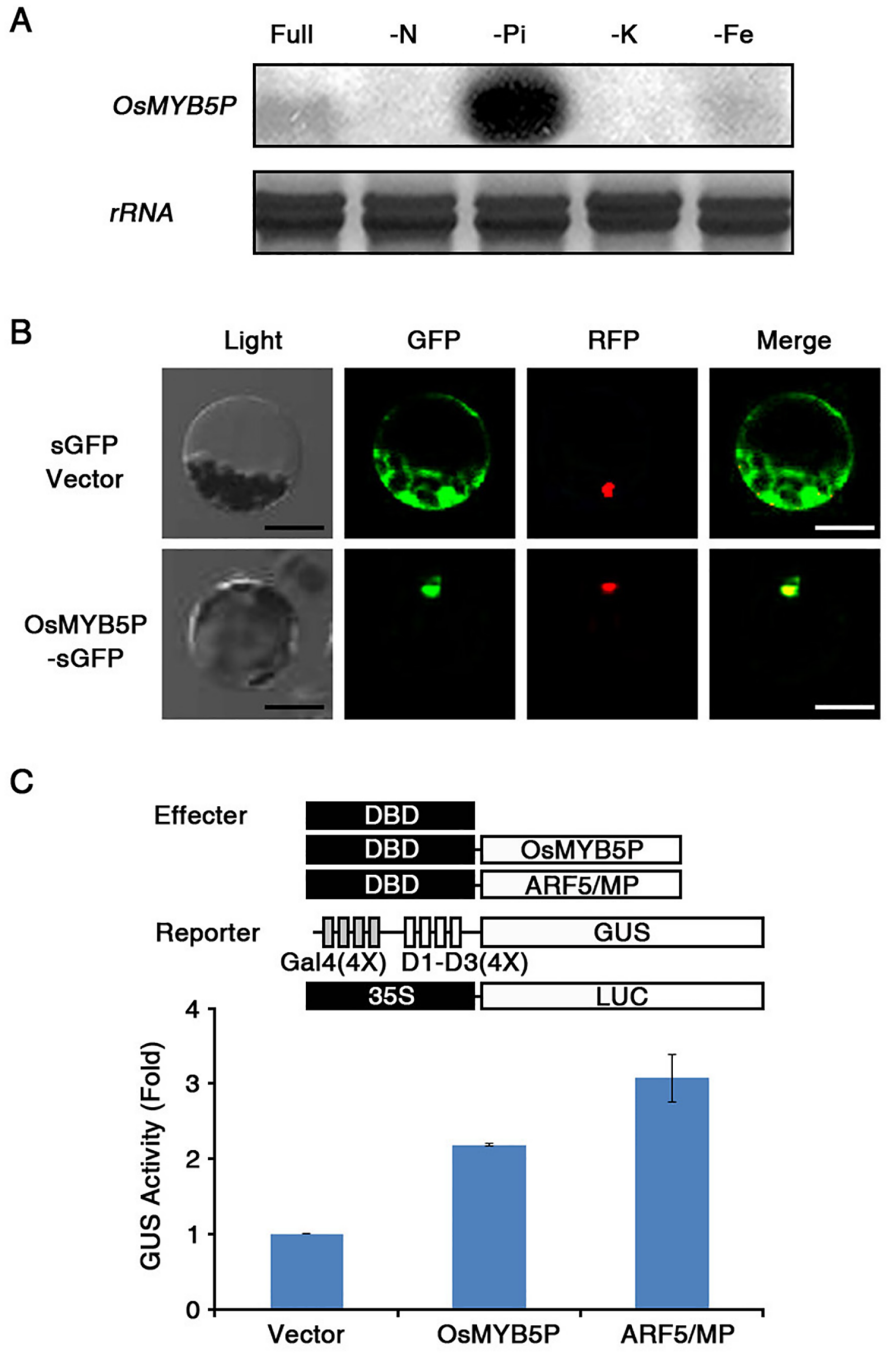
Functional characterization of *OsMYB5P*. (A) Expression of an *OsMYB5P* transcript in rice suspension cells under nutrient-deficient conditions. Rice suspension cells were transferred to nitrogen (N; 0.25 mM), phosphate (Pi; 0.0125 mM), potassium (K; 0.01 mM), or iron (Fe; 0.01 mM)-deficient media for 6 h. Total RNA was extracted from these nutrient-deprived cells. rRNA is a loading control. (B) Subcellular localization of OsMYB5P using *Arabidopsis* protoplast systems. *Arabidopsis* protoplasts were transiently co-transformed with a *CaMV35S*:*OsMYB5P-sGFP* or *CaMV35S*:*sGFP* vector and *NLS-RFP* constructs. *NLS-RFP* was used as a nuclear marker protein. After 24 h, GFP and RFP signals of transformed protoplasts were monitored using a fluorescence microscope. Scale bar indicates 20 μm. (C) Transcriptional activity assay of *OsMYB5P*. A schematic representation (top) shows the effector and reporter constructs used in the transient expression assay. *Arabidopsis* protoplasts were co-transfected using combinations with each effector along with two reporter constructs. *ARF5/MP* was used as a positive control, and *35S*:*LUC* was used as an internal control. After normalization to LUC activity, GUS activity in each sample was measured. Bars represent the mean ± standard deviation of three technical replicates.

It has recently been reported that MYB transcription factors in rice are key regulators for phosphate acquisition in phosphate starvation signaling [37, 38]. To investigate how rice MYB transcription factors respond to phosphate starvation, we isolated and identified an R2R3-type MYB transcription factor, OsMYB5P (*Os02g0624300*), from rice. OsMYB5P encodes an R2R3-type MYB transcription factor that is encoded by nucleotide 774 bp and includes 258 amino acids with a total molecular mass of approximately 28.49 kDa (http://rapdb.dna.affrc.go.jp/). To find out whether the transcription level of *OsMYB5P* is specifically regulated by nutrient-limited conditions, we transferred cell suspensions from rice to different nutrient-deficient media for 6 h. Northern blot analysis showed that the expression of *OsMYB5P* was highly induced by Pi-deficiency stress (Fig 1A). In contrast, *OsMYB5P* was either weakly expressed or not expressed at all in response to deprivation of other nutrients (N, K, and Fe).

To find out whether OsMYB5P is localized in the nucleus like other transcription factors, we fused the full-length cDNA of *OsMYB5P* to the N-terminus of sGFP protein under the control of a *35S* promoter. The fluorescence signal of the *OsMYB5P-sGFP* was detected primarily in the cell nucleus, whereas that of the *sGFP* vector alone was distributed throughout the cytoplasm (Fig 1B). For the nuclear marker, we used a chimeric construct containing nuclear localization signal (NLS) proteins fused to red fluorescent protein (RFP). To analyze the transcriptional activation ability of OsMYB5P, we performed a transient expression assay in *Arabidopsis* protoplasts. *OsMYB5P* fused to the yeast *GAL4* DNA binding domain (DBD) effector and a constitutively expressed reporter gene, including four upstream *GAL4* DNA binding sites (GAL4[4X]-D1-D3[4X]-GUS), was co-transfected into *Arabidopsis* protoplasts (Fig 1C). We used the empty vector (Vector) or DBD:ARF5/MP (ARF5/MP) as negative or positive control [38, 44]. As expected, *OsMYB5P* strongly increased GUS activity, much more than the vector alone. These results indicated that transcription of *OsMYB5P* is highly induced under Pi-deficient conditions, and OsMYB5P can function as a transcriptional activator in the nucleus.

### OsMYB5P modulates plant development and Pi uptake in rice

**Fig 2:**
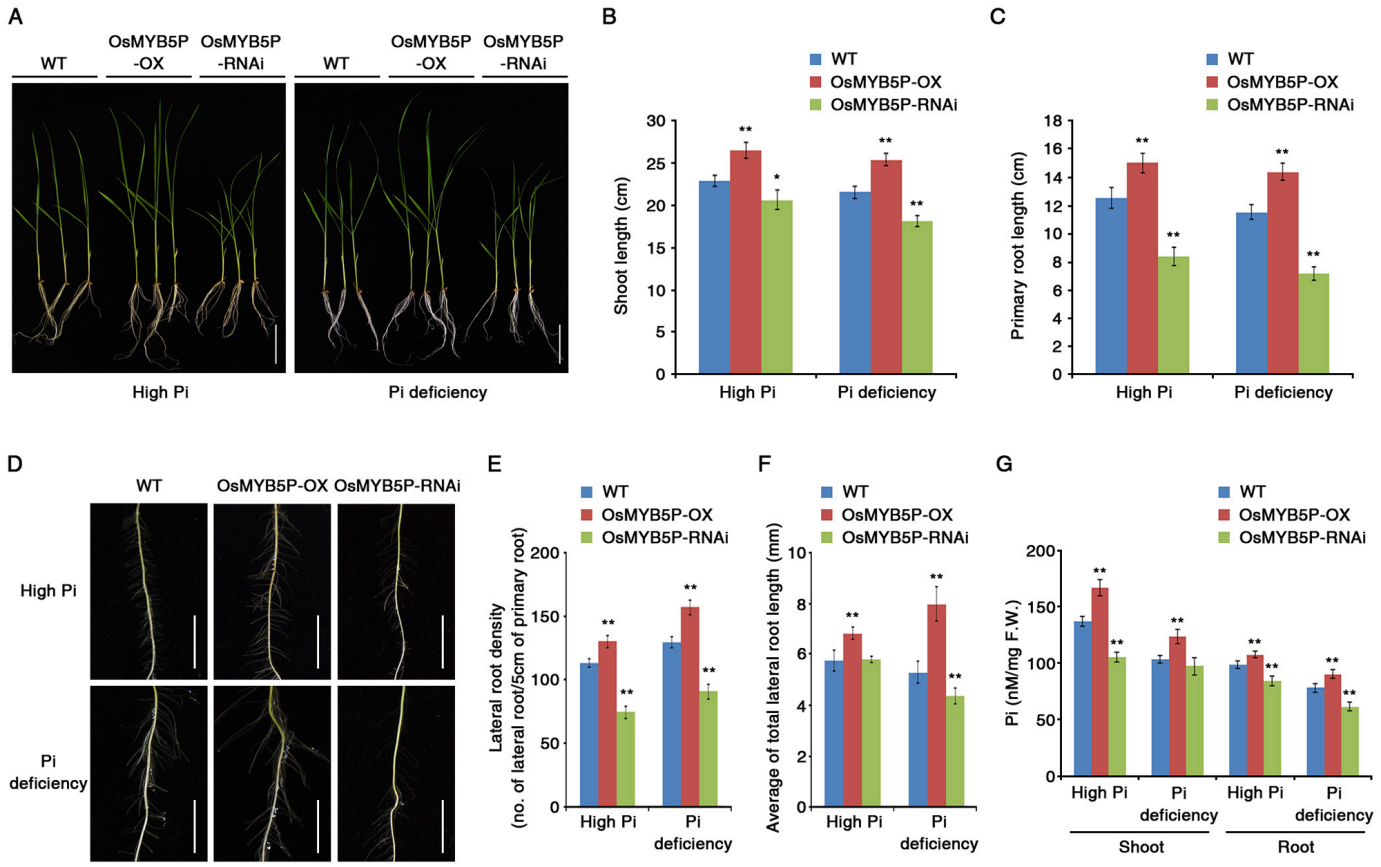
Physiological characterization of OsMYB5P responses to Pi deficiency. (A) Seven-day-old WT, OsMYB5P-OX, and OsMYB5P-RNAi seedlings were grown vertically for 7 d on high Pi (1.25 mM KH_2_PO_4_) or Pi-deficient (0.0125 mM KH_2_PO_4_) media. Scale bar indicates 5 cm. (B and C) Graphical representation of the shoot (B) and primary root (C) length of seedlings depicted in (A). Error bars represent mean ± SD of n = 10 replicates of 3 seedlings for each experiment. (D) Lateral root development at the tip of the primary root of plants depicted in (A). Scale bar indicates 1 cm. (E and F) Graphical representation of the lateral root densities (E) or total lateral root lengths (F) of primary root of plants shown in (A). Measurement of density or average is the number or length of lateral roots along 5 cm of root above the root tip. Error bars represent mean ± SD of n = 10 replicates of 3 seedlings for each experiment. (G) Inorganic Pi concentrations were measured in the shoots and roots of plants under both high Pi and Pi-deficient conditions. Error bars represent mean ± SD of n = 6 replicates of 10 seedlings for each experiment. Asterisks represent significant differences from the WT (*, 0.01 < p-value ≤ 0.05; **, p-value ˂ 0.01; Student’s *t*-test).

To functionally characterize *OsMYB5P* in response and adaptation to Pi deprivation, we generated two transgenic plants with contrasting expression patterns in rice: *OsMYB5P* overexpression (OsMYB5P-OX) and RNA interference (OsMYB5P-RNAi) lines (S1A and S2A Figs). We detected transcription of OsMYB5P in OsMYB5P-OX and OsMYB5P-RNAi transgenic plants under a high concentration of Pi (High Pi, 1.25 mM) or a low concentration of Pi (Pi deficiency, 0.0125 mM) by qRT-PCR (S3 Fig). When 7-d-old OsMYB5P-OX, OsMYB5P-RNAi, and WT plants were exposed to culture media containing either high Pi or low Pi for 7 d, both the shoots and the roots of OsMYB5P-OX plants accumulated higher biomass than did the shoots and roots of WT plants, but those of OsMYB5P-RNAi plants did not (Fig 2A) (S1, S2 and S4 Figs). In addition, the shoots and primary roots of OsMYB5P-OX plants grew better under high-Pi and Pi-deficient conditions than did shoots and primary roots of WT plants (Fig 2B and 2C). When these transgenic plants were grown for 3 weeks, plant growth was similar to that of growth for only 7 d between transgenic plants and WT (S6 Fig). Lateral root density and growth of lateral roots were significantly higher in OsMYB5P-OX plants than in WT plants grown in either high-Pi or Pi-deficient conditions (Fig 2D–2F).

To understand the function of OsMYB5P in Pi uptake, we measured the Pi concentration of OsMYB5P-OX, OsMYB5P-RNAi, and WT plants under high-Pi or Pi-deficient conditions (Fig 2G) (S1E, S2E and S6D Figs). The Pi concentration in shoots and roots of OsMYB5P-OX plants was higher than that of WT plants grown under high Pi or under Pi deficiency. In contrast, the Pi in OsMYB5P-RNAi plants was maintained at a relatively lower level than in WT plants. Taken together, these results suggest that OsMYB5P may play a regulatory role in Pi uptake and adaptation to Pi-deficiency stress.

### OsMYB5P is associated with OsPT5 gene expression through MBS motifs

**Fig 3:**
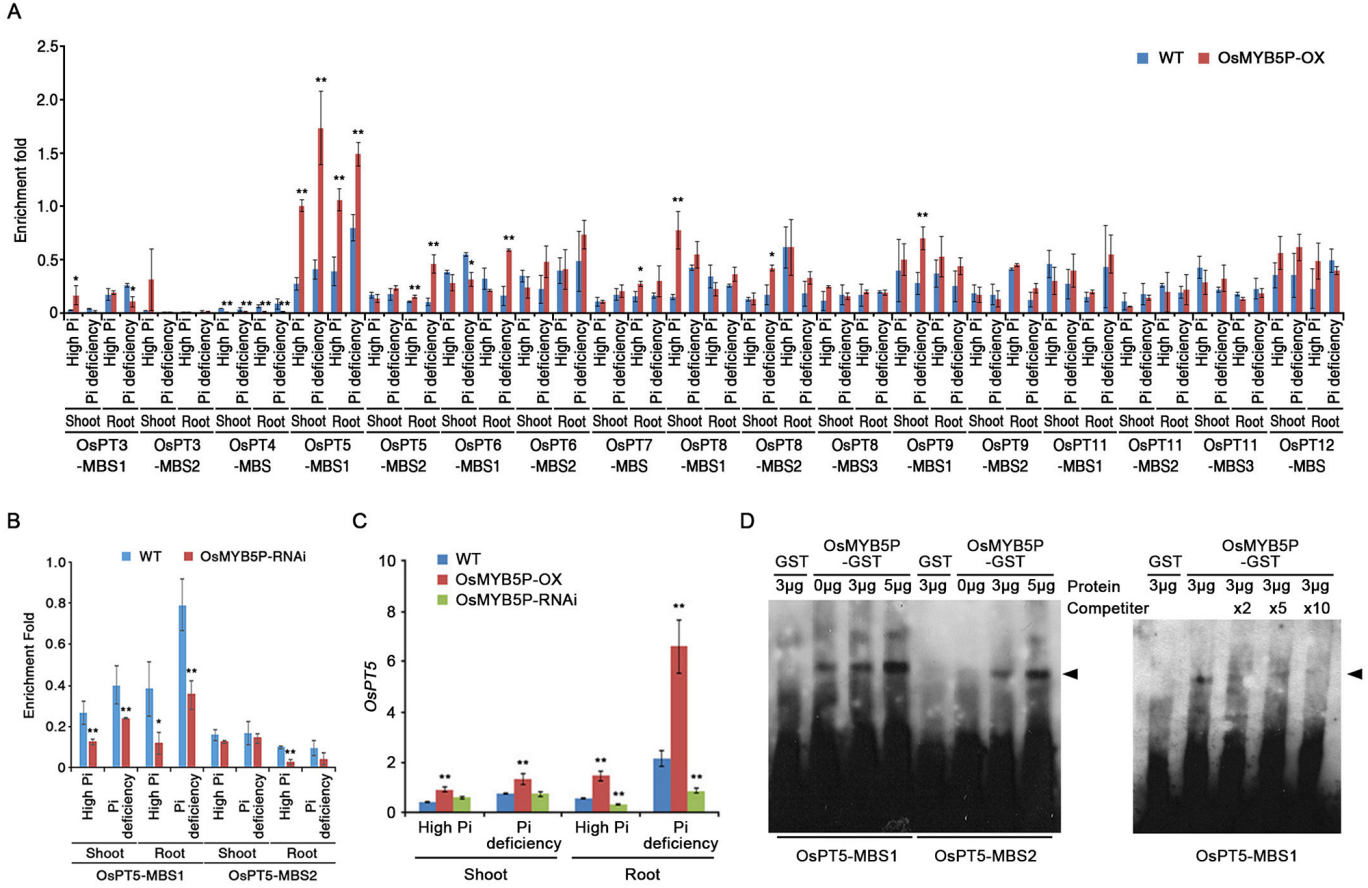
OsMYB5P associated with *OsPT5*. (A) ChIP assay of OsMYB5P binding to the OsPTs promoter. Seven-day-old WT and OsMYB5P-OX seedlings were grown vertically for 7 d on high Pi or Pi deficient media. Then, the shoots and roots were harvested separately for the ChIP assay with anti-OsMYB5P. Fold enrichment of the ratio of OsMYB5P-OX to WT signal is shown here. Error bars represent the mean ± SD of three technical replicates. (B) OsMYB5P associated with OsPT5 in OsMYB5P-RNAi plants. ChIP assay was used to detect the association between OsMYB5P and the *OsPT5* promoter. Seven-day-old WT and OsMYB5P-RNAi seedlings were grown vertically for 7 d on high Pi or Pi deficient media. Then, the shoots and roots were harvested separately for the ChIP assay with anti-OsMYB5P. Fold enrichment of the ratio of OsMYB5P-RNAi to WT signal is shown here. Error bars represent the mean ± SD of three technical replicates. (C) qRT-PCR analysis of the relative expression levels of the *OsPT5* gene in shoots and roots of seedlings grown under high Pi or Pi-deficient conditions. Expression levels of *OsACTIN1* were used for normalization. Error bars represent the mean ± SD of three technical replicates. Asterisks represent significant differences from the WT (*, 0.01 < p-value ≤ 0.05; **, p-value < 0.01; Student’s *t*-test). (D) EMSA was used to analyze the binding of OsMYB5P to MBS fragments (MBS1 and MBS2) of the *OsPT5* promoter. The DNA probes containing the MBS motif were amplified using biotin-labeled (left) or cold-probe (right; competitor). The labeled DNA-OsMYB5P complex is indicated by an arrow head.

Pi-responsive transporters increase plant growth and development by helping to maintain Pi homeostasis. In particular, most *PTs* induce transcriptional expression of many Pi starvation-inducible (PSI) genes by Pi starvation [26, 27]. To investigate how the transcription factor OsMYB5P is involved in *OsPTs* gene expression, we performed an *in silico* analysis of the presumptive promoter region using the PlantCARE database (http://bioinformatics.psb.ugent.be/webtools/plantcare/html/). Several *OsPTs* promoters include putative *cis*-acting regulatory elements of the MYB binding site (MBS), which is typically associated with abiotic stress responses (S1 Table). To identify MBSs associated with *OsPTs* based on the *in silico* analysis, we performed a chromatin immunoprecipitation (ChIP) assay using nucleus protein extracts of WT and OsMYB5P-OX transgenic plants. After immunoprecipitation with an antiserum against OsMYB5P (Fig 3A and 3B) (S7B Fig), the relative content of MBS fragments in the *OsPTs* promoter was estimated by qRT-PCR. Interestingly, the amplicons *OsPT5*-MBS1 and *OsPT5*-MBS2 in the MBS region of OsMYB5P-OX plants were significantly more enriched than those in the WT (Fig 3A). Moreover, enrichment of the amplicons *OsPT5*-MBS1 in OsMYB5P-RNAi plants was significantly reduced more than that of the WT (Fig 3B). No enrichment of other MBS regions in other *OsPTs* promoters was observed in the OsMYB5P-OX extracts, except for *OsPT5* (Fig 3A). To test whether *OsMYB5P* plays a role in *OsPT5*-mediated Pi-starvation signaling, we compared the expression of *OsPT5* mRNA transcripts in WT, OsMYB5P-OX, and OsMYB5P-RNAi plants in high-Pi and Pi-deficient conditions by qRT-PCR. Significant increases in steady-state levels of *OsPT5* transcripts were observed in shoots and roots of OsMYB5P-OX plants (Fig 3C). However, the *OsPT5* transcript was more weakly expressed in OsMYB5P-RNAi than in the WT. These results suggest that *OsMYB5P* is involved in *OsPT5*-mediated Pi-deficiency signaling and Pi uptake in rice.

To examine whether OsMYB5P protein binds to one or both of these MBSs in the *OsPT5* promoter, we performed an electrophoretic mobility shift assay (EMSA) with biotin-labeled oligonucleotides corresponding to promoter fragments containing MBS motifs (*OsPT5*-MBS1 and *OsPT5*-MBS2) and recombinant OsMYB5P-GST or GST proteins (S7A Fig). A GST-OsMYB5P-specific mobility-retarded band indicating binding to OsMYB5P was observed with the *OsPT5*-MBS1 oligonucleotide (Fig 3D, left). The intensity of this band was enhanced by increasing the amount of GST-OsMYB5P protein in the binding reaction. OsMYB5P mobility-retarded bands were observed with the *OsPT5*-MBS2 oligonucleotide, indicating weak binding (Fig 3D, left). Moreover, OsMYB5P/*OsPT5*-MBS1 mobility-retarded bands were observed with competitor oligonucleotides, indicating decreased binding (Fig 3D, right). Together, the results from the EMSA and ChIP assays indicate that OsMYB5P directly binds to MBS regions in the *OsPT5* promoter *in vitro* and *in vivo*.

### Absence of OsPT5 affects Pi-starvation responses in rice

**Fig 4:**
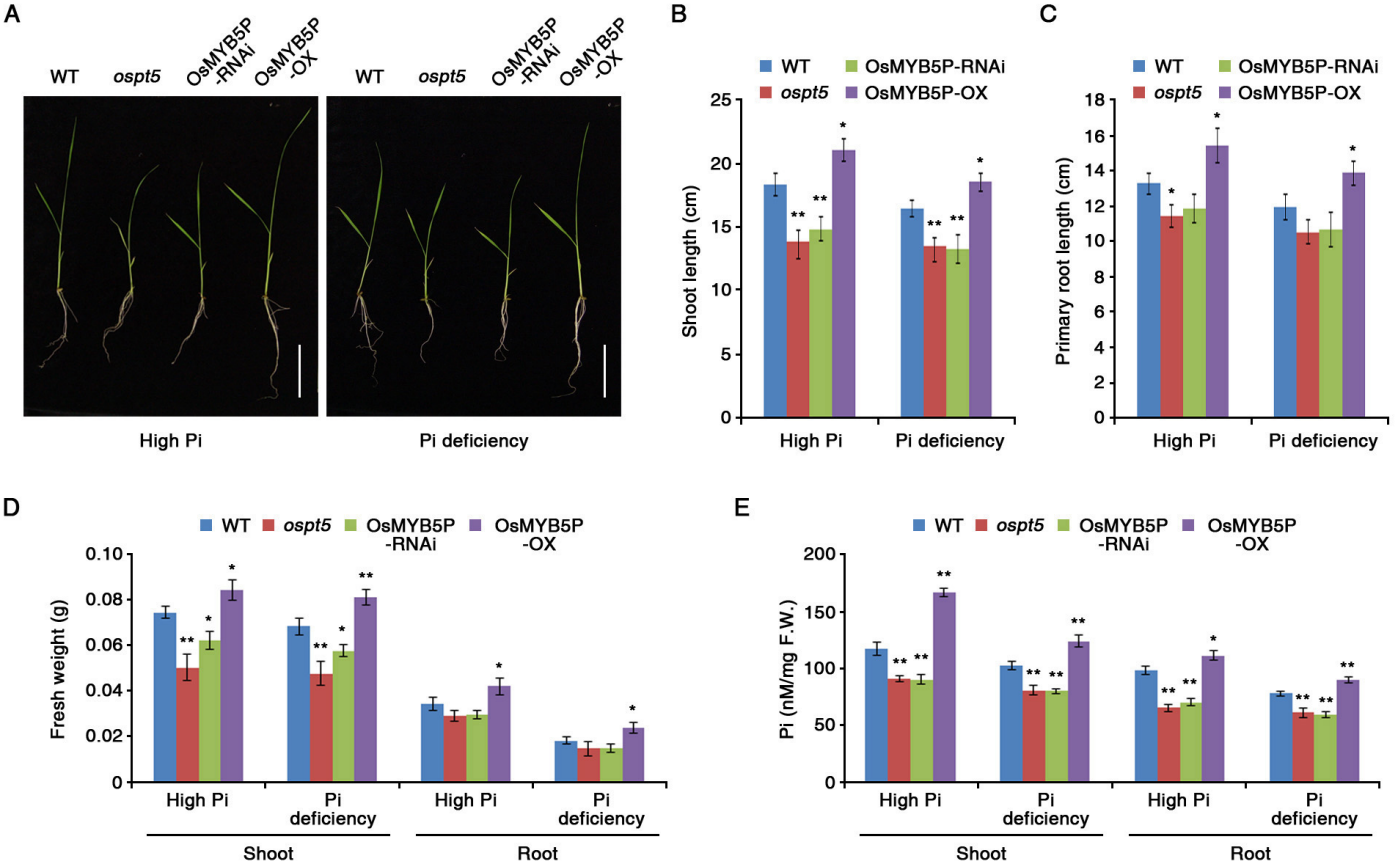
Physiological characterization of *ospt5* mutant under Pi deficiency. (A) Seven-day-old WT, *ospt5*, OsMYB5P-OX, and OsMYB5P-RNAi seedlings were grown vertically for 7 d on high Pi (1.25 mM KH_2_PO_4_) or Pi-deficient (0.0125 mM KH_2_PO_4_) media. Scale bar indicates 5 cm. (B and C) Graphical representation of the shoot (B) and primary root (C) length of seedlings depicted in (A). Error bars represent mean ± SD of n = 10 replicates of 3 seedlings for each experiment. (D) Fresh shoot and root biomass of *ospt5* mutant plants. Seven-day-old seedlings were grown for 7 d in high Pi or Pi-deficient media, after which shoots and roots were sampled separately. Error bars represent mean ± SD of n = 10 replicates of 3 seedlings for each experiment. (E) Inorganic Pi concentrations were measured in the shoots and roots of plants under both high Pi and Pi-deficient conditions. Error bars represent mean ± SD of n = 6 replicates of 10 seedlings for each experiment. Asterisks represent significant differences from the WT (*, 0.01 < p-value ≤ 0.05; **, p-value < 0.01; Student’s *t*-test).

Although functions of some OsPTs were reported in Pi-starvation responses [20, 23, 37], the function of *OsPT5* was still unknown. To prove the above hypothesis, we acquired an *ospt5* mutant that contains a T-DNA insertion in the first exon of *OsPT5* (S5 Fig; Dr. Jung kindly provided the rice *ospt5* mutant plants). Like the phenotype of OsMYB5P-RNAi plants, growth of the shoot and primary root in the *ospt5* mutant was more inhibited than in WT plants in high-Pi and Pi-deficient media (Fig 4A–4C). Moreover, the fresh weights of *ospt5* and OsMYB5P-RNAi plants were comparable (Fig 4D). Furthermore, there was no difference in the Pi content of roots and shoots of *ospt5* and OsMYB5P-RNAi plants grown in high-Pi and Pi-deficient media (Fig 4E). As the absence of *OsPT5* produced the expected phenotypes, we conclude that OsMYB5P and OsPT5 play an important role for maintaining Pi homeostasis in Pi starvation signaling.

### OsMYB5P affects expression of rice PSI genes

**Fig 5:**
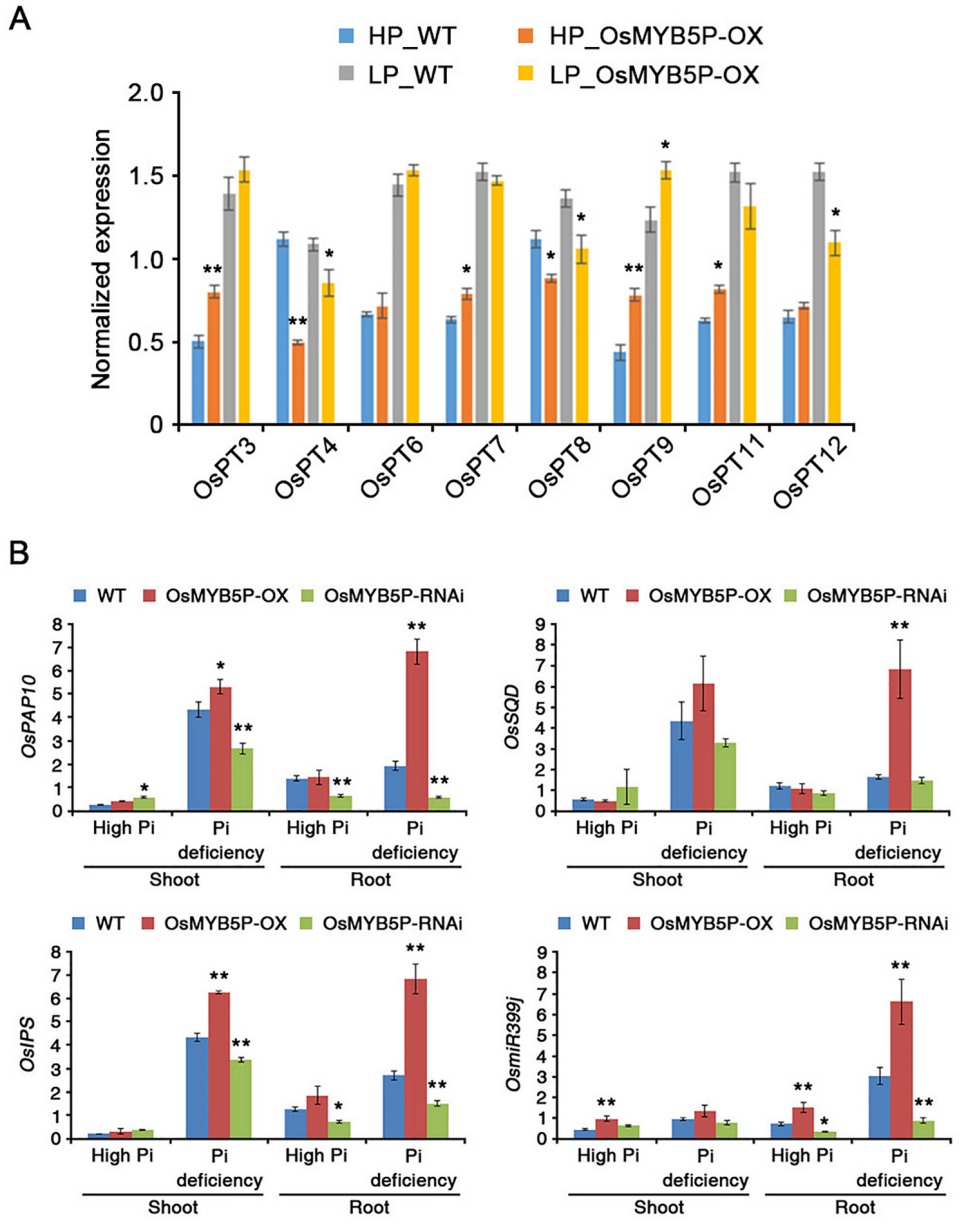
Expression of PSI genes in WT and OsMYB5P transgenic plants. (A) Expression of *OsPTs* genes in the WT and OsMYB5P-OX transgenic plants. Total RNA was extracted from WT and OsMYB5P-OX seedlings grown under high Pi (HP) or Pi deficient (LP) conditions. (B) Expression of *OsPAP10*, *OsSQD*, *OsIPS*, and *OsmiR399j* genes in the WT and OsMYB5P-OX transgenic plants. Total RNA was extracted from shoots and roots of seedlings grown under high Pi or Pi-deficient conditions. Expression of *OsACTIN1* was used for normalization. Error bars represent mean ± SD of three technical replicates. Asterisks represent significant differences from the WT (*, 0.01 < p-value ≤ 0.05; **, p-value < 0.01; Student’s *t*-test).

*In silico* analysis showed that promoter regions of most *OsPTs*, except *OsPT1*, *OsPT2* and *OsPT10*, included several putative MBS *cis*-acting elements (S1 Table). We showed evidence that transcript of *OsPT5* was significantly increased by overexpressing of OsMYB5P (Fig 3C). However, although some *OsPTs*, *OsPT3* and *OsPT9*, were highly expressed in OsMYB5P-OX plants (Fig 5A), one cannot conclude that transcript of these genes was directly regulated by OsMYB5P. Transcriptional expression of many PSR genes in downstream of signaling pathway affects plant developmental and physiological responses during Pi starvation [27, 45]. Our data show that overexpression of *OsMYB5P* induces plant growth under both normal Pi and Pi-deficient conditions (Fig 2). To validate whether *OsMYB5P* changes the expression of rice PSI genes, we investigated the transcript levels of several genes, including *OsPAP10*, *OsSQD*, *OsIPS*, and *OsmiR399j*, in the shoots and roots of WT, OsMYB5P-OX, and OsMYB5P-RNAi plants (Fig 5B). Compared to the WT, transcript levels of most PSI genes were high in the shoots and roots of OsMYB5P-OX plants under Pi-deficient conditions. In contrast, transcripts of most PSI genes were downregulated in both shoots and roots of OsMYB5P-RNAi plants more than in the WT under Pi-deficient conditions. These data suggest that OsMYB5P regulates transcript levels of PSI genes to maintain Pi homeostasis in rice under Pi deprivation. Interestingly, however, expression of these genes in OsMYB5P-OX and OsMYB5P-RNAi plants showed no change under high-Pi conditions (Fig 5B). These results supported the evidence that Pi accumulation by OsMYB5P promoted plant growth during high-Pi conditions (Fig 2), unrelated expression of PSI genes.

### Overexpression of OsMYB5P increases Pi accumulation and Pi-starvation responses in transgenic *Arabidopsis* plants

**Fig 6:**
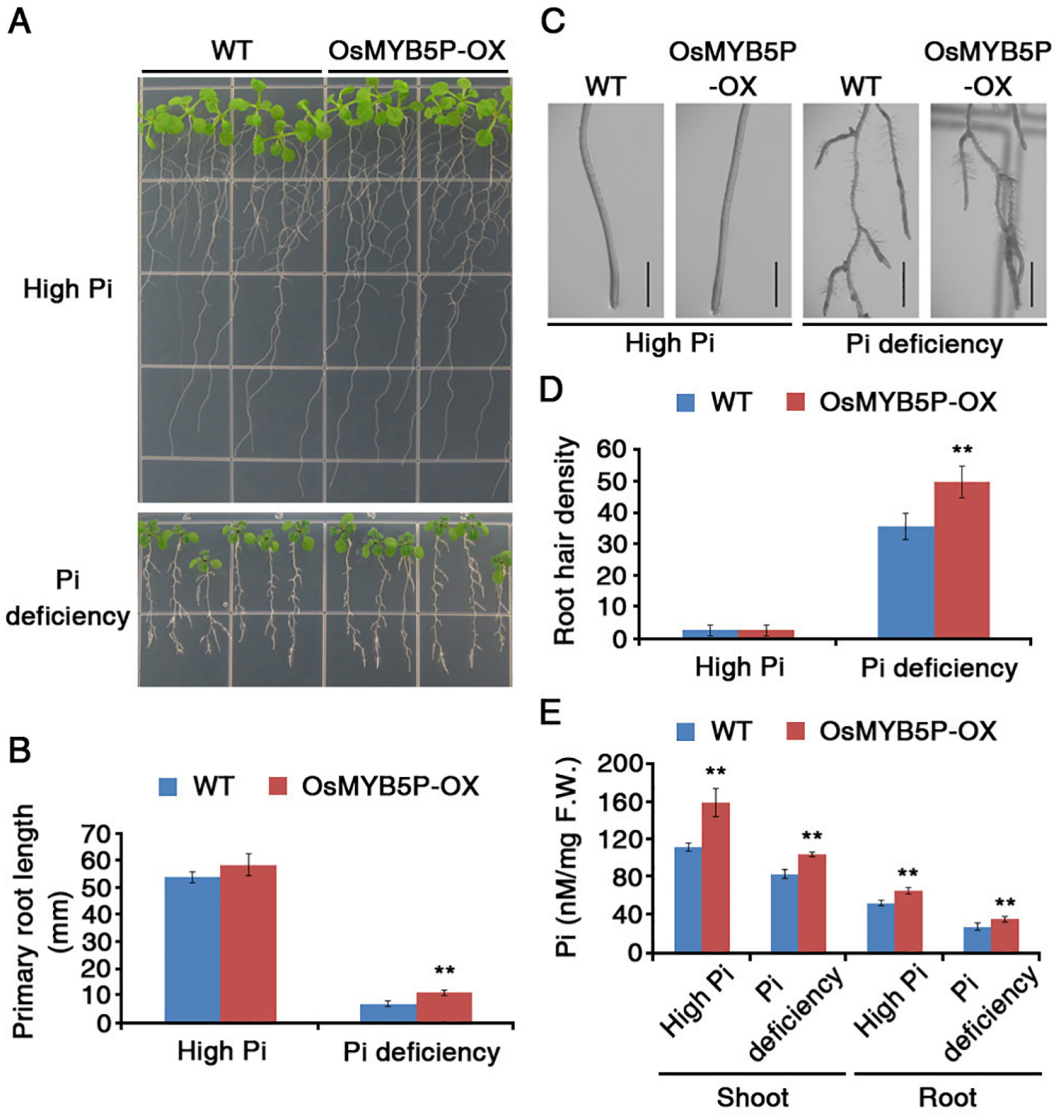
Phenotypic alteration in root architecture of *Arabidopsis* OsMYB5P transgenic plants. (A) Four-day-old *Arabidopsis* WT and OsMYB5P-OX seedlings were grown vertically for 7 d on high Pi (1.25 mM KH_2_PO_4_) or Pi-deficient (0 mM KH_2_PO_4_) media. (B) Quantification of primary root lengths of the seedlings depicted in (A). Error bars represent mean ± SD of n = 6 replicates of 18 seedlings for each experiment. (C) Comparison of root architectures of *Arabidopsis* between WT and OsMYB5P-OX seedlings depicted in (A). Scale bar indicates 1 mm. (D) Quantification of root hair densities at the primary root tip of plants shown in (C). Root hair density was calculated as the number of root hairs along 5 mm of each root above the tip. Error bars represent mean ± SD of n = 6 replicates of 18 seedlings for each experiment. (E) Inorganic Pi concentrations were measured in the shoots and roots of *Arabidopsis* seedlings. Error bars represent mean ± SD of n = 6 replicates of 18 seedlings for each experiment. Asterisks represent significant differences from the WT (*, 0.01 < p-value ≤ 0.05; **, p-value < 0.01; Student’s *t*-test). F.W. indicates fresh weight.

To understand the possible roles of OsMYB5P in plant Pi-starvation responses, *OsMYB5P* was overexpressed in *Arabidopsis* WT plants under the control of the *CaMV* 35S promoter (S8A Fig). To test for *OsMYB5P* expression, we used northern blot analysis in transgenic *Arabidopsis* plants to generate T3 homozygous lines for further analysis (S8B Fig). To investigate whether overexpression of *OsMYB5P* correlated with the Pi-starvation response in both monocots and dicots, we examined the phenotype of *OsMYB5P* transgenic *Arabidopsis* plants under Pi-deficient conditions, as with the rice OsMYB5P-OX plants (Fig 2). Under high-Pi conditions, no obvious difference in the phenotype between *Arabidopsis* WT and transgenic *Arabidopsis* plants was observed (Fig 6). After Pi deprivation, the primary roots of *OsMYB5P* transgenic *Arabidopsis* plants grew slightly larger than those of *Arabidopsis* WT plants (Fig 6A and 6B). However, there were more lateral roots and denser root hairs in *OsMYB5P* transgenic *Arabidopsis* plants than in *Arabidopsis* WT plants, with the exception of lateral root length (Fig 6C and 6D) (S9 Fig). In addition, Pi accumulated more in both shoots and roots of *OsMYB5P* transgenic *Arabidopsis* plants than in those of WT *Arabidopsis* plants (Fig 6E). These results indicate that overexpression of *OsMYB5P* increases tolerance to Pi deprivation and promotes Pi accumulation during Pi-deficient conditions in *Arabidopsis*.

### OsMYB5P regulates expression of *Arabidopsis* phosphate transporters

**Fig 7:**
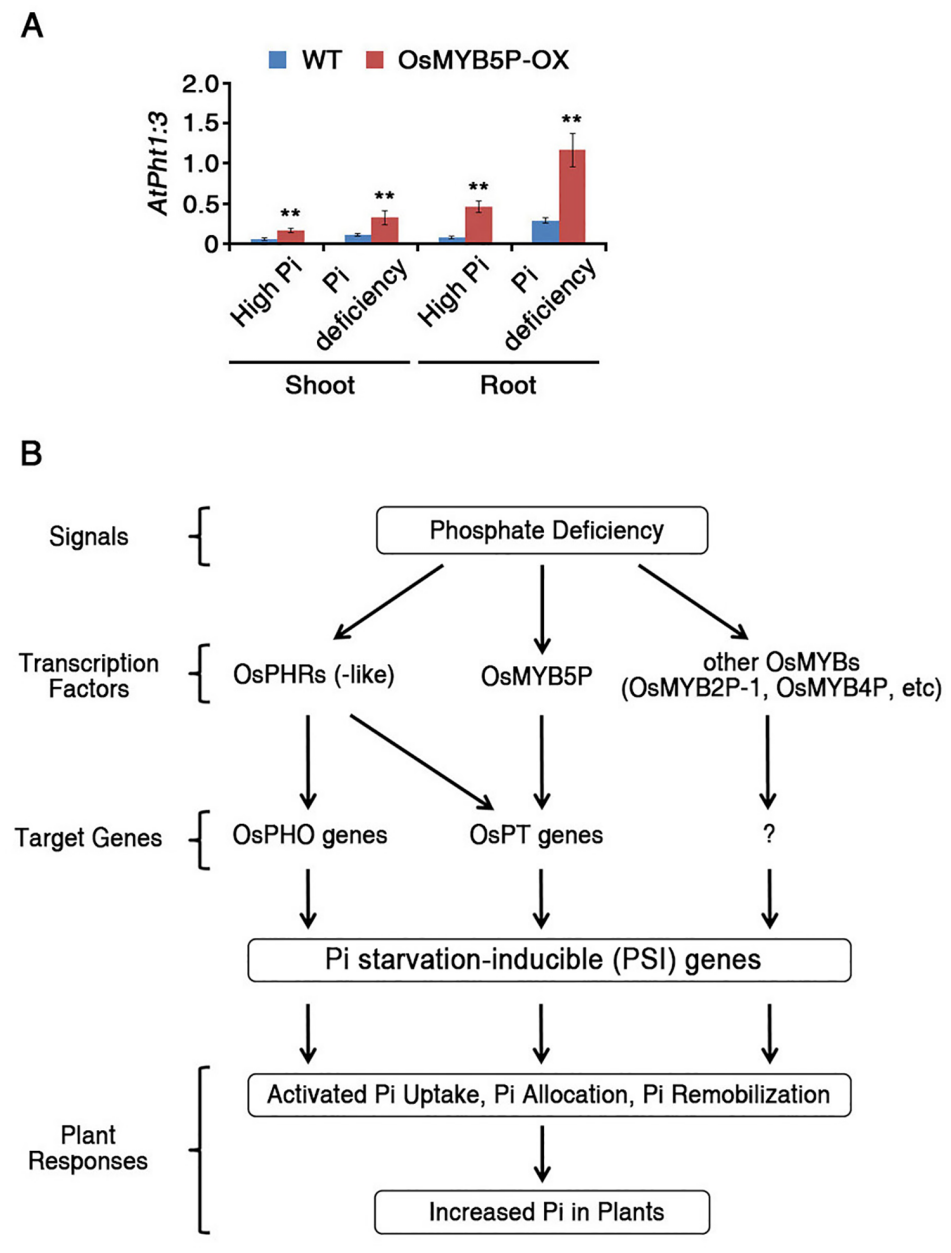
Expression of *Arabidopsis* PTs in *Arabidopsis* WT and OsMYB5P transgenic plants. (A) Total RNA was extracted from shoots and roots of *Arabidopsis* WT and OsMYB5P-OX seedlings grown under high Pi or Pi-deficient conditions. Expression levels of *AtTUBULIN2* were used for normalization. Bars represent the mean ± standard error of three technical replicates. Asterisks represent significant differences from the WT (*, 0.01 < p-value ≤ 0.05; **, p-value < 0.01; Student’s *t*-test). (B) Working model of the role of OsMYB5P in various Pi starvation responses.

In *Arabidopsis*, the *Pht1* family contains nine members, *AtPht1;1* to *AtPht1;9*, which play major roles in the regulation of phosphate uptake in Pi acquisition and translocation [13]. Four additional major phosphate transporters involved in the *Arabidopsis* Pi-starvation response are also known from other families: *AtPht2;1*, *AtPht3;1*, *AtPht3;2*, and *AtPht3;3* [46, 47]. Previous experimental data demonstrate that *OsMYB5P* directly regulates phosphate transporters through binding at the promoter in rice (Fig 3). To investigate whether *OsMYB5P* regulates *Arabidopsis* phosphate transporters, we performed qRT-PCR analysis in shoots and roots under Pi-deficient conditions. Among the *Arabidopsis* phosphate transporters, transcripts of *AtPht1*:*3* were more highly induced in both shoots and roots of *OsMYB5P* transgenic *Arabidopsis* plants under both high-Pi and Pi-deficient conditions than in *Arabidopsis* WT plants (Fig 7A). The *AtPht1;3* promoter contains one MBS *cis*-element as identified by *in silico* analysis (S2 Table). However, expression levels of other phosphate transporters in both shoots and roots of *OsMYB5P* transgenic *Arabidopsis* plants were similar to those of *Arabidopsis* WT plants under high-Pi and Pi-deficient conditions (S10 Fig). Although several other factors regulate phosphate transporters during Pi-starvation responses, these data indicate that transcripts of *AtPht1*:*3* are highly expressed in *OsMYB5P* transgenic *Arabidopsis* plants, providing a molecular basis upon which *OsMYB5P* might modulate the expression of phosphate uptake signaling at the transcriptional level.

## Discussion

The Pi-starvation response in plants is induced by various phenomena, including physiological and morphological processes, and involves the transcriptional expression of numerous genes [48]. Although many studies of the transcription factors involved in this response have been conducted, current knowledge of the function of MYB transcription factors is limited. In this study, we isolated and identified *OsMYB5P*, a rice gene encoding a novel R2R3-type MYB transcription factor, and characterized its role in the Pi-starvation response by overexpression and RNAi knock-down with *OsMYB5P* in monocots and dicots. We demonstrated that *OsMYB5P* plays a regulatory role in the transcriptional expression of a Pi transporter and contributes to Pi acquisition to maintain Pi homeostasis in rice and *Arabidopsis*.

Various specific and general MYB transcription factors have been shown to regulate the progression of Pi-starvation responses in monocots and dicots [48–50]. The major transcription factors are activated by the signals of Pi starvation when plants are exposed to Pi-deficiency stress and respond by various mechanisms, such as activated Pi uptake, Pi allocation, and Pi remobilization (Fig 7B). *PHR1*, a well-known transcription factor in the CC-type MYB family, is an important regulator in the Pi-starvation response that controls Pi homeostasis by enhancing *PHO2* cleavage by *miR399* [51]. OsPHR2 directly regulates *OsPT2* transcriptional expression under Pi-deficient conditions [52]. Meanwhile, other MYB transcription factors, such as OsMYB2P-1 and OsMYB4P, showed no evidence directly regulation of PSI genes. However, they suggested indirectly evidence that changed the transcript levels of PSI genes to accumulate Pi (Fig 7B) [37, 38]. In our study, we suggest that *OsMYB5P*, a member of the R2R3-type MYB family in rice, directly and specifically induces the transcription of Pi transporters, especially *OsPT5* and *AtPht1;3*, in both shoots and roots (Fig 7B).

### Specific function of OsMYB5P in Pi homeostasis in rice

The MYB transcription factors, particularly the R2R3-type, act as important regulators in developmental growth, signaling transduction, abiotic stress, and nutrient-deficiency tolerance [53–55]. The R2R3-MYB transcription factors include 88 proteins, and they localize to the nucleus in rice [55]. The roles of R2R3-MYB transcription factors in the maintenance of Pi homeostasis in rice are poorly understood. At present, we know that two R2R3-MYB transcription factors (of 88 in the rice R2R3-MYB family) play a key regulatory role in the Pi-starvation response [37, 38]. We established that *OsMYB5P*, like *OsMYB2P-1* and *OsMYB4P*, consists of R2R3-MYB domains at the N-terminus and localizes to the nucleus (Fig 1B) (S11 Fig). It has been reported that expression of *OsMYB2P-1* and *OsMYB4P* is induced in shoots and roots when they are deprived of Pi and other nutrients [37, 38]. We demonstrated that *OsMYB5P* is also highly expressed in shoots and roots during Pi deprivation (Fig 1A). In addition to the R2R3-MYB family, *OsPHR1* and *OsPHR2*, genes that encode transcription factors in the MYB-CC family, are involved in Pi- and Fe-starvation responses in rice [26, 32]. Thus, to our knowledge, the evidence presented herein is the first report to show that *OsMYB5P* is related to the regulation of a strictly Pi-starvation response regardless of other nutrient levels in rice.

### Overexpression of OsMYB5P confers tolerance to Pi deprivation in plants

Many transcription factors in rice have been shown to be involved in signal transduction and regulation through control of gene expression induced by the Pi-starvation response [48, 56]. Some responses function like other regulatory mechanisms in rice. For example, overexpression of *OsPHR1*, *OsPHR2*, and *OsMYB2P-1* genes retards plant growth by Pi accumulation under normal conditions [27, 32, 37]. However, *OsMYB2P-1* overexpression increases shoot and root growth under Pi-deficient conditions [37], and overexpression of *OsMYB4P* promotes plant growth in both normal and Pi-deficient conditions [38]. In our experiments, shoots and roots in *OsMYB5P* overexpressing transgenic plants were found to grow better than the WT under normal conditions, whereas *OsMYB5P* RNAi plants showed decreased growth in shoots and roots (Fig 2). Interestingly, our data demonstrated that overexpression of OsMYB5P was not an effect of the most *OsPTs* genes expression, but highly induced the expression of *OsPT5* in both shoots and roots during high-Pi conditions (Figs 3 and 5). Therefore, we suggested that promotion of plant growth by overexpressing of OsMYB5P was causative of *OsPT5* level, which regulates the acquisition and transport Pi in plants.

Transgenic plants that overexpress OsMYB5P tolerated Pi deficiency better, as indicated by increased shoot and root biomass (Fig 2) (S4 and S6 Figs). Moreover, the increased tolerance of Pi-deficiency stress by *OsMYB5P*-overexpressing plants occurs simultaneously with increasing expression of PSI genes, including *OsPAP10a*, *OsSQD*, *OsIPS*, and *OsmiR399j* (Fig 5B). *OsPAP10a* and *OsSQD* encode an acid phosphatase and sulfolipid synthase, respectively, and are involved in the Pi-starvation response [32, 57]. *OsmiR399* and *OsIPS* play a critical role in controlling plant Pi uptake [26, 58–60]. Activation of acid phosphatases and scavenging systems in Pi-starvation responses is a functional adaptation to make the best use of Pi availability for plants under Pi-deficient conditions [4, 61]. OsPHR2, a central regulator in Pi starvation response, overexpressing were not only up-regulated some PSI genes, but also increased Pi accumulation in both shoots and roots [32]. Like OsPHR2 overexpressing, OsMYB5P overexpressing plants was similar observed in our results. Thus, our evidences suggested that OsMYB5P was an important regulator in Pi uptake and Pi translocation, although transcript levels of *OsPT5* and PSI genes by OsMYB5P were highly increased in roots compared to those in shoots during Pi deficiency (Figs 3C and 5B). In addition, the increased tolerance by *OsMYB5P* transgenic plants of Pi deficiency may be associated with altered expression patterns of PSI genes.

### OsMYB5P is a positive regulator that maintains Pi homeostasis in plants

Pi transporters are directly responsible for Pi acquisition and transport in plants [13, 62]. OsPHR2 directly regulates *OsPT2* transcription by binding to P1BS (PHR1 binding sequence; *GNATATNC*) on the promoter [63]. Although most PTs in rice contain the P1BS motif in the promoter, only *OsPT2* is regulated by OsPHR2 in Pi-starvation responses [52]. We demonstrated the biological evidence to prove these hypotheses by ChIP assay (Fig 3A). Our study revealed that *OsPT5* may, in part, account for the observed Pi uptake in shoots and roots of *OsMYB5P*-overexpressing transgenic plants under Pi-deficient conditions, as evidenced by the greater upregulation of this gene in such plants than in the WT (Fig 3C). Overexpression of *OsMYB5P* resulted in higher Pi content in transgenic rice plants than in WT plants under Pi-deficient conditions (Fig 2G). In addition, Pi content in *ospt5* mutant plants was lower than in WT plants, as it also was in OsMYB5P-RNAi plants (Fig 4E). Therefore, *OsMYB5P* may regulate Pi acquisition by targeting *OsPT5* at the transcriptional level. Furthermore, overexpression of *OsMYB5P* in *Arabidopsis* increases transcriptional levels of Pi transporters *AtPht1*:*3* (Fig 7A). Finally, our results suggest that *OsMYB5P* is likely to play a role in the positive regulation of Pi-dependent transporters, which in turn may facilitate Pi acquisition under Pi-deficient conditions in both monocots and dicots.

## Conclusions

In summary, this study characterized an OsMYB5P transcription factor belonging to the R2R3-type of the MYB family that is localized to the nucleus and acts as a transcriptional activator. OsMYB5P acts as an important regulator of Pi-starvation responses, such that overexpression of *OsMYB5P* results in a larger root-system architecture and more shoot development under normal and Pi-deficient conditions, increased tolerance to Pi-deficiency stress, and improved expression of PSI genes. In addition, our results demonstrate that *OsMYB5P* can function as a positive regulator of Pi transporters in monocots and dicots.

## Supporting information

S1 Fig Physiological phenotypes of OsMYB5P-OX plants under Pi deficiency. (A) Transcriptional expression of *OsMYB5P* in different OsMYB5P-OX transgenic plants. Total RNA was extracted from WT and three independent OsMYB5P-OX seedlings grown under high Pi conditions. Expression of *OsACTIN1* was used for normalization. Error bars represent the mean ± SD of three technical replicates. Asterisks represent significant differences from the WT (*; 0.01 < p-value ≤ 0.05, **; p-value ˂ 0.01, Student’s t-test). (B) Seven-day-old WT, and three independent OsMYB5P-OX seedlings were grown vertically for 7 d on high Pi (1.25 mM KH_2_PO_4_) or Pi deficient (0.0125 mM KH_2_PO_4_) media. Scale bar indicates 5 cm. (C and D) Graphical representation of the shoot (C) or primary root (D) length of seedlings depicted in (B). Error bars represent mean ± SD of n = 10 replicates of 3 seedlings for each experiment. (E) Inorganic Pi concentrations were measured in the shoots and roots of plants under both high Pi and Pi deficient conditions. Error bars represent mean ± SD of n = 6 replicates of 10 seedlings for each experiment. Asterisks represent significant differences from the WT (*; 0.01 < p-value ≤ 0.05, **; p-value < 0.01, Student’s t-test).
(TIF)

S2 Fig Physiological phenotypes of OsMYB5P-RNAi plants to Pi deficiency. (A) Transcriptional expression of *OsMYB5P* in different OsMYB5P-RNAi transgenic plants by northern blot analysis. Total RNA was extracted from WT and three independent OsMYB5P-RNAi seedlings grown under high Pi conditions. rRNA used a loading control. (B) Seven-day-old WT, and three independent OsMYB5P-RNAi seedlings were grown vertically for 7 d on high Pi (1.25 mM KH_2_PO_4_) or Pi deficient (0.0125 mM KH_2_PO_4_) media. Scale bar indicates 5 cm. (C and D) Graphical representation of the shoot (C) or primary root (D) length of seedlings depicted in (B). Error bars represent mean ± SD of n = 10 replicates of 3 seedlings for each experiment. (E) Inorganic Pi concentrations were measured in the shoots and roots of plants under both high Pi and Pi deficient conditions. Error bars represent mean ± SD of n = 6 replicates of 10 seedlings for each experiment. Asterisks represent significant differences from the WT (*; 0.01 < p-value ≤ 0.05, **; p-value < 0.01, Student’s t-test).
(TIF)

S3 Fig Expression of *OsMYB5P* in WT and OsMYB5P transgenic plants. Total RNA was extracted from shoots and roots of seedlings grown under high Pi or Pi deficient conditions. Expression of *OsACTIN1* was used for normalization. Error bars represent the mean ± SD of three technical replicates. Asterisks represent significant differences from the WT (*; 0.01 < p-value ≤ 0.05, **; p-value < 0.01, Student’s t-test).
(TIF)

S4 Fig Biomass analysis of OsMYB5P transgenic plants to Pi deficiency. Seven-day-old seedlings were grown for 7 d in high Pi or Pi deficient media, after which shoots (A) and roots (B) were sampled separately. Error bars represent mean ± SD of n = 10 replicates of 3 seedlings for each experiment. Asterisks represent significant differences from the WT (**; p-value < 0.01, Student’s t-test).
(TIF)

S5 Fig Characterization of *ospt5* mutant in rice. (A) Schematic illustration is a representation of the location of T-DNA insertions in *ospt5* mutant. (B) Total RNA was extracted from shoots and roots of *ospt5* mutant grown under high Pi conditions. Expression of *OsACTIN1* was used for normalization. Error bars represent the mean ± SD of three technical replicates. Asterisks represent significant differences from the WT (**; p-value < 0.01, Student’s t-test).
(TIF)

S6 Fig Physiological phenotypes of OsMYB5P-OX, OsMYB5P-RNAi and *ospt5* plants to Pi deficiency. (A) Seven-day-old WT, OsMYB5P-OX, OsMYB5P-RNAi and *ospt5* seedlings were grown vertically for 3 weeks on high Pi (1.25 mM KH_2_PO_4_) or Pi deficient (0.0125 mM KH_2_PO_4_) media. Scale bar indicates 5 cm. (B and C) Graphical representation of the shoot (B) or primary root (C) length of seedlings depicted in (A). Error bars represent mean ± SD of n = 10 replicates of 3 seedlings for each experiment. (D) Inorganic Pi concentrations were measured in the shoots and roots of plants under both high Pi and Pi deficient conditions. Error bars represent mean ± SD of n = 6 replicates of 10 seedlings for each experiment. Asterisks represent significant differences from the WT (*; 0.01 < p-value ≤ 0.05, **; p-value < 0.01, Student’s t-test).
(TIF)

S7 Fig OsMYB5P associated with OsPT5 in OsMYB5P-RNAi plants. (A) Induction of OsMYB5P protein from *Escherichia coli* using 0.5 mM IPTG at 30 °C for 3 h. The SDS-PAGE gel with GST and OsMYB5P-GST proteins was stained using Coomassie brilliant blue. M denotes a protein size marker. (B) Immunoprecipitation assay with mouse anti-OsMYB5P antibody. To detect the endogenous OsMYB5P in rice, total proteins were extracted from rice WT, OsMYB5P-OX, and OsMYB5P-RNAi plants, and then immunoprecipitated with mouse anti-OsMYB5P monoclonal antibody. The arrow and arrowhead indicate endogenous OsMYB5P protein (lower panel; approximately 28.49 kDa) and a non-specific band (upper panel), respectively.
(TIF)

S8 Fig Expression of *OsMYB5P* in *Arabidopsis* OsMYB5P transgenic plants. (A) Schematic diagram of the *OsMYB5P* chimeric plasmid structure. The full-length cDNA of *OsMYB5P* is under the control of a *35S* promoter, and linked to the hygromycine resistance gene (*hpt*) and the green fluorescent protein (E*gfp*). (B) Expression of *OsMYB5P* in *Arabidopsis OsMYB5P* transgenic plants by northern blot analysis. Total RNA was extracted from 11 representative transgenic lines. *rRNA* is a loading control.
(TIF)

S9 Fig Development of lateral roots in *Arabidopsis* OsMYB5P transgenic plants during Pi deficiency. Total lateral root number (A) or length (B) on primary root of plants shown in Fig 5C. Error bars represent the mean ± SD of n = 6 replicates with 18 seedlings for each experiment. Asterisks represent significant differences from the WT (*; 0.01< p-value ≤ 0.05, **; p-value ≤ 0.01, Student’s t-test).
(TIF)

S10 Fig Expression of *Arabidopsis* PT genes in the *Arabidopsis* WT and OsMYB5P transgenic plants. Total RNA was extracted from shoots and roots of *Arabidopsis* WT and OsMYB5P-OX seedlings grown under high Pi or Pi deficient conditions. Expression levels of *AtTUBULIN2* were used for normalization. Bars represent the mean ± standard error of three technical replicates. Asterisks represent significant differences from the WT (*; 0.01 < p-value ≤ 0.05, **; p-value < 0.01, Student’s t-test).
(TIF)

S11 Fig Schematic structure of OsMYB5P. The two types of MYB domain repeats on the N terminus of OsMYB5P are indicated with red (R2) and blue (R3). The alignment of the DNA-binding (R2 and R3) domains with the amino acid sequence of rice R2R3-MYB transcription factors involved in Pi starvation responses was performed using CLUSTAL W. Identical amino acids are shaded black, and similar amino acids are shaded in gray.
(TIF)

S1 Table Putative MBS *cis*-elements in the promoter of rice phosphate transporters (*OsPTs*). (DOCX)

S2 Table Putative MBS *cis*-elements in the promoter of *Arabidopsis* phosphate transporters (*AtPhts*). (DOCX)

S3 Table Primer lists for our studies. (DOCX)
