# Supplementary figures and images for: Rice OsMYB5P improves plant phosphate acquisition by regulation of phosphate transporter
Source: PLoS One. 2018 Mar 22;13(3):e0194628. doi: 10.1371/journal.pone.0194628 (PMC5864048; doi:10.1371/journal.pone.0194628)

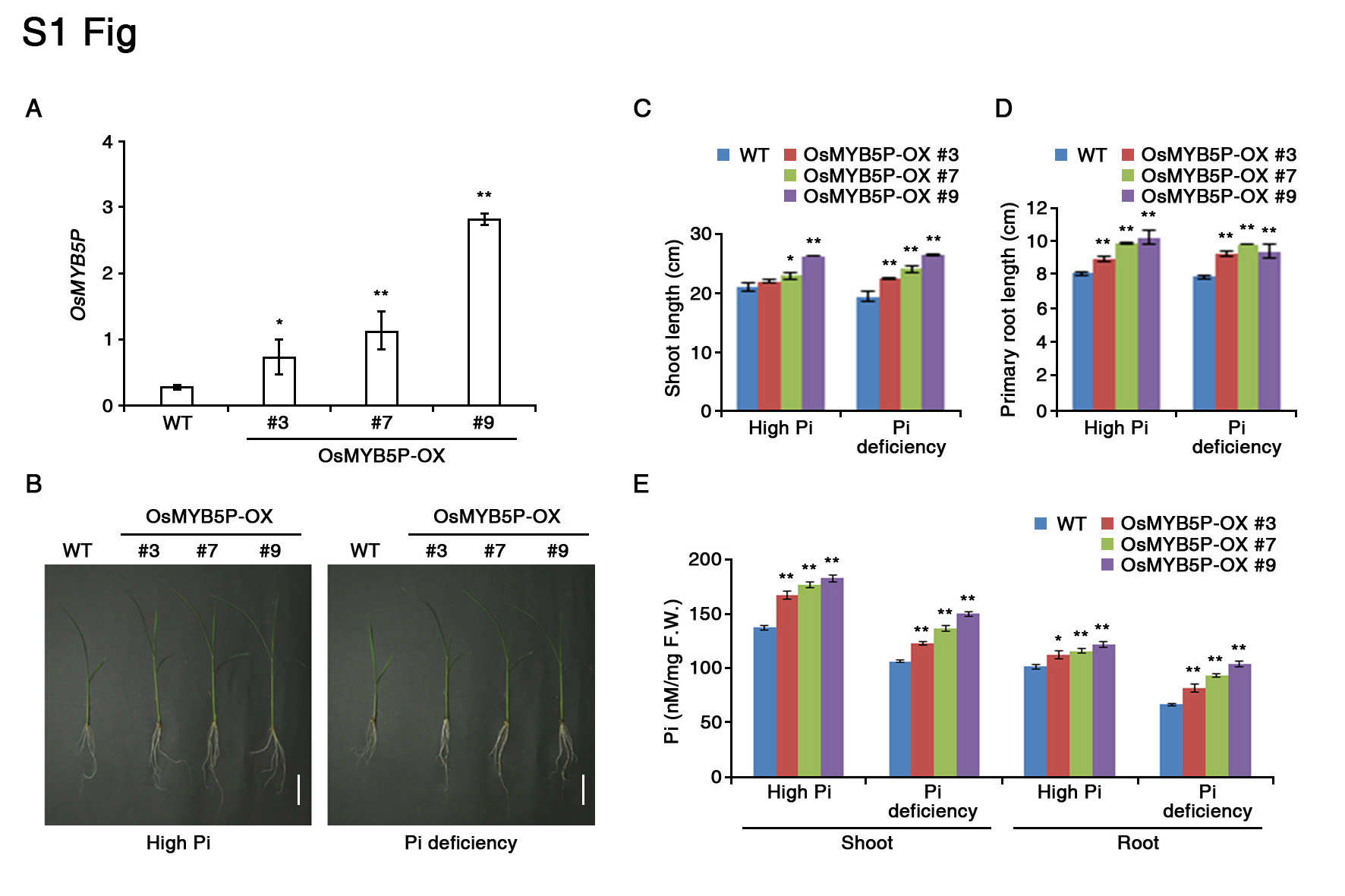

Supplement: S1 Fig — (A) Transcriptional expression of OsMYB5P in different OsMYB5P-OX transgenic plants. Total RNA was extracted from WT and three independent OsMYB5P-OX seedlings grown under high Pi conditions. Expression of OsACTIN1 was used for normalization. Error bars represent the mean ± SD of three technical replicates. Asterisks represent significant differences from the WT (*; 0.01 < p-value ≤ 0.05, **; p-value ˂ 0.01, Student’s t-test). (B) Seven-day-old WT, and three independent OsMYB5P-OX seedlings were grown vertically for 7 d on high Pi (1.25 mM KH2PO4) or Pi deficient (0.0125 mM KH2PO4) media. Scale bar indicates 5 cm. (C and D) Graphical representation of the shoot (C) or primary root (D) length of seedlings depicted in (B). Error bars represent mean ± SD of n = 10 replicates of 3 seedlings for each experiment. (E) Inorganic Pi concentrations were measured in the shoots and roots of plants under both high Pi and Pi deficient conditions. Error bars represent mean ± SD of n = 6 replicates of 10 seedlings for each experiment. Asterisks represent significant differences from the WT (*; 0.01 < p-value ≤ 0.05, **; p-value < 0.01, Student’s t-test). (TIF) [file pone.0194628.s001.tif]

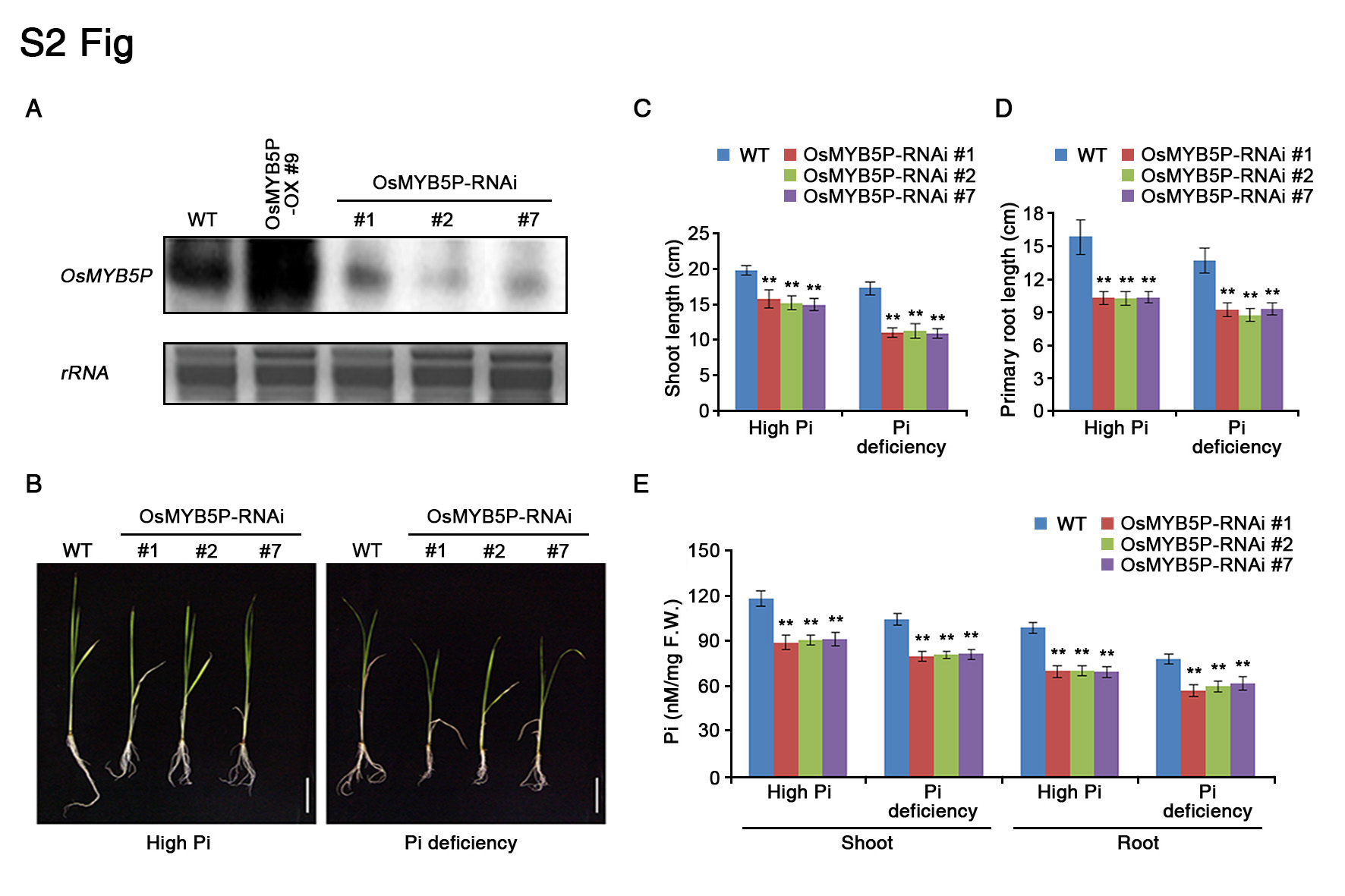

Supplement: S2 Fig — (A) Transcriptional expression of OsMYB5P in different OsMYB5P-RNAi transgenic plants by northern blot analysis. Total RNA was extracted from WT and three independent OsMYB5P-RNAi seedlings grown under high Pi conditions. rRNA used a loading control. (B) Seven-day-old WT, and three independent OsMYB5P-RNAi seedlings were grown vertically for 7 d on high Pi (1.25 mM KH2PO4) or Pi deficient (0.0125 mM KH2PO4) media. Scale bar indicates 5 cm. (C and D) Graphical representation of the shoot (C) or primary root (D) length of seedlings depicted in (B). Error bars represent mean ± SD of n = 10 replicates of 3 seedlings for each experiment. (E) Inorganic Pi concentrations were measured in the shoots and roots of plants under both high Pi and Pi deficient conditions. Error bars represent mean ± SD of n = 6 replicates of 10 seedlings for each experiment. Asterisks represent significant differences from the WT (*; 0.01 < p-value ≤ 0.05, **; p-value < 0.01, Student’s t-test). (TIF) [file pone.0194628.s002.tif]

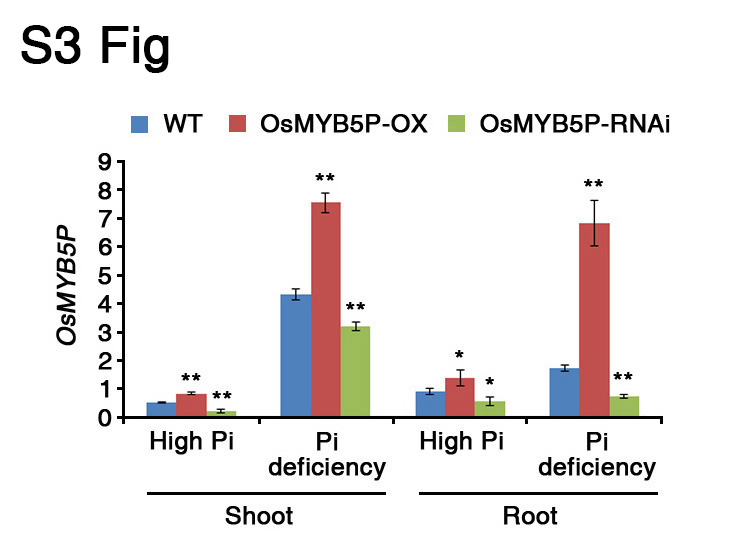

Supplement: S3 Fig — Total RNA was extracted from shoots and roots of seedlings grown under high Pi or Pi deficient conditions. Expression of OsACTIN1 was used for normalization. Error bars represent the mean ± SD of three technical replicates. Asterisks represent significant differences from the WT (*; 0.01 < p-value ≤ 0.05, **; p-value < 0.01, Student’s t-test). (TIF) [file pone.0194628.s003.tif]

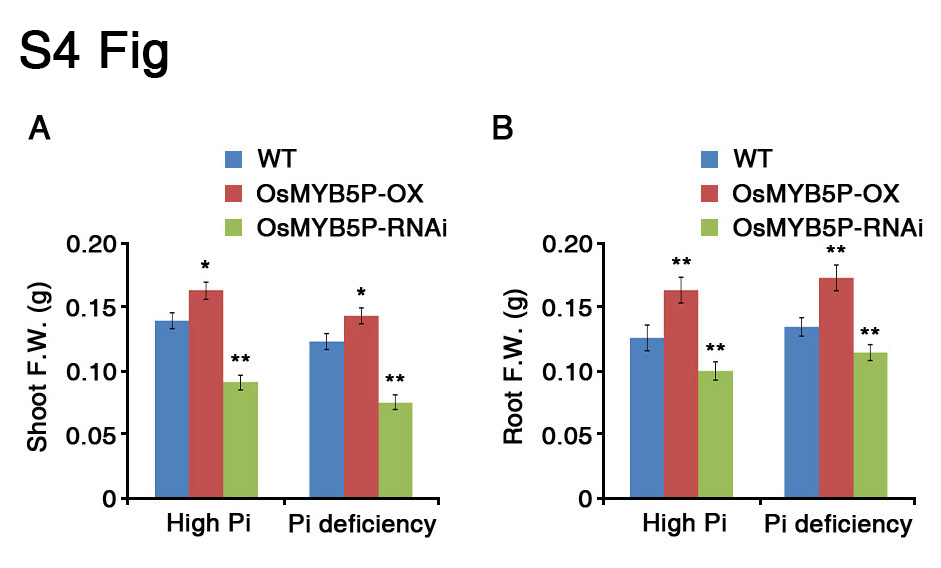

Supplement: S4 Fig — Seven-day-old seedlings were grown for 7 d in high Pi or Pi deficient media, after which shoots (A) and roots (B) were sampled separately. Error bars represent mean ± SD of n = 10 replicates of 3 seedlings for each experiment. Asterisks represent significant differences from the WT (**; p-value < 0.01, Student’s t-test). (TIF) [file pone.0194628.s004.tif]

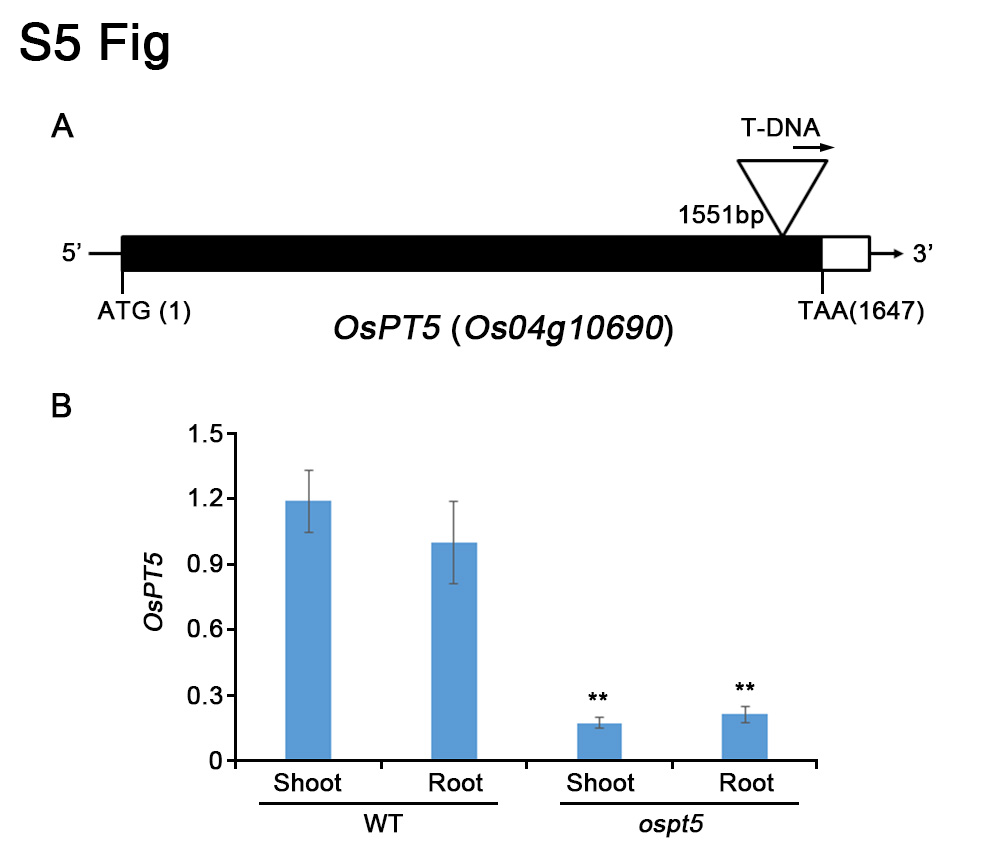

Supplement: S5 Fig — (A) Schematic illustration is a representation of the location of T-DNA insertions in ospt5 mutant. (B) Total RNA was extracted from shoots and roots of ospt5 mutant grown under high Pi conditions. Expression of OsACTIN1 was used for normalization. Error bars represent the mean ± SD of three technical replicates. Asterisks represent significant differences from the WT (**; p-value < 0.01, Student’s t-test). (TIF) [file pone.0194628.s005.tif]

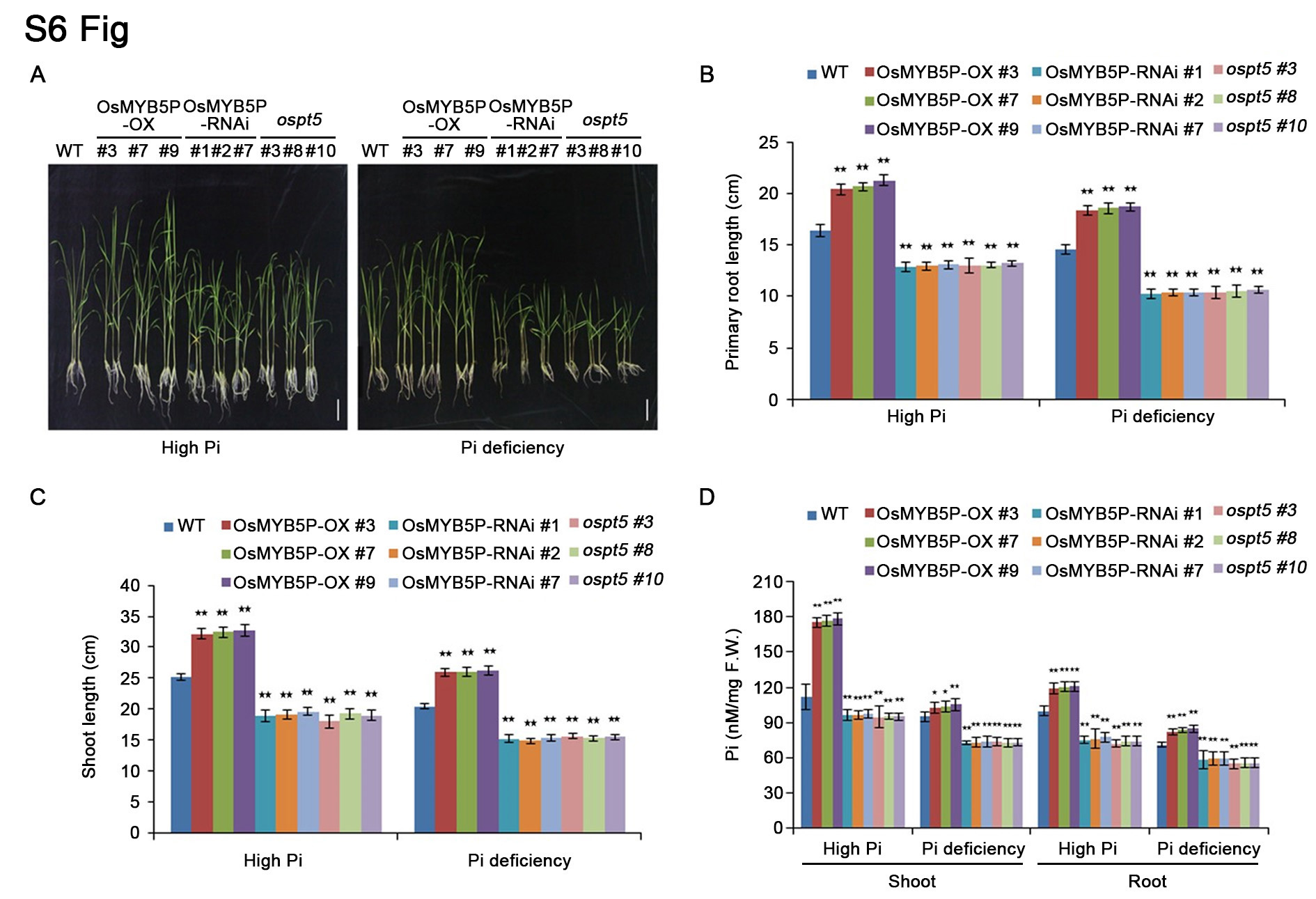

Supplement: S6 Fig — (A) Seven-day-old WT, OsMYB5P-OX, OsMYB5P-RNAi and ospt5 seedlings were grown vertically for 3 weeks on high Pi (1.25 mM KH2PO4) or Pi deficient (0.0125 mM KH2PO4) media. Scale bar indicates 5 cm. (B and C) Graphical representation of the shoot (B) or primary root (C) length of seedlings depicted in (A). Error bars represent mean ± SD of n = 10 replicates of 3 seedlings for each experiment. (D) Inorganic Pi concentrations were measured in the shoots and roots of plants under both high Pi and Pi deficient conditions. Error bars represent mean ± SD of n = 6 replicates of 10 seedlings for each experiment. Asterisks represent significant differences from the WT (*; 0.01 < p-value ≤ 0.05, **; p-value < 0.01, Student’s t-test). (TIF) [file pone.0194628.s006.tif]

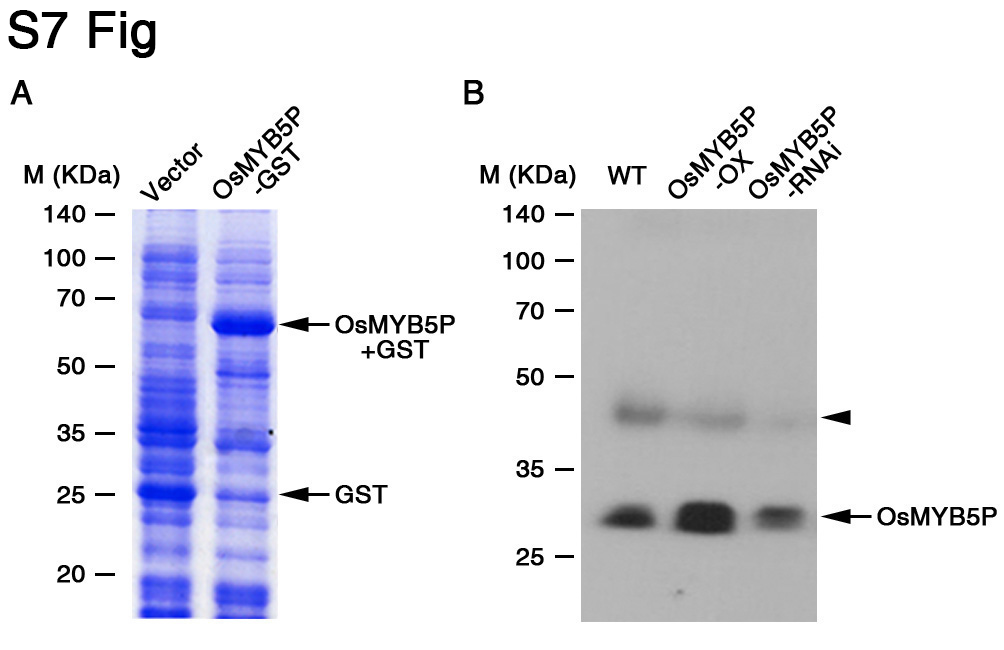

Supplement: S7 Fig — (A) Induction of OsMYB5P protein from Escherichia coli using 0.5 mM IPTG at 30 °C for 3 h. The SDS-PAGE gel with GST and OsMYB5P-GST proteins was stained using Coomassie brilliant blue. M denotes a protein size marker. (B) Immunoprecipitation assay with mouse anti-OsMYB5P antibody. To detect the endogenous OsMYB5P in rice, total proteins were extracted from rice WT, OsMYB5P-OX, and OsMYB5P-RNAi plants, and then immunoprecipitated with mouse anti-OsMYB5P monoclonal antibody. The arrow and arrowhead indicate endogenous OsMYB5P protein (lower panel; approximately 28.49 kDa) and a non-specific band (upper panel), respectively. (TIF) [file pone.0194628.s007.tif]

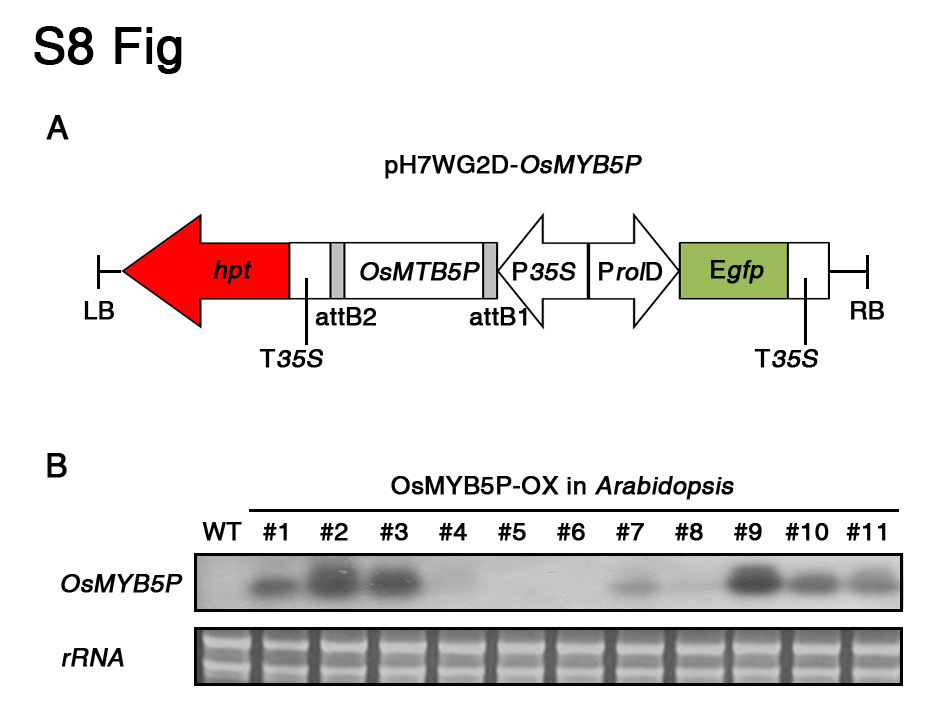

Supplement: S8 Fig — (A) Schematic diagram of the OsMYB5P chimeric plasmid structure. The full-length cDNA of OsMYB5P is under the control of a 35S promoter, and linked to the hygromycine resistance gene (hpt) and the green fluorescent protein (Egfp). (B) Expression of OsMYB5P in Arabidopsis OsMYB5P transgenic plants by northern blot analysis. Total RNA was extracted from 11 representative transgenic lines. rRNA is a loading control. (TIF) [file pone.0194628.s008.tif]

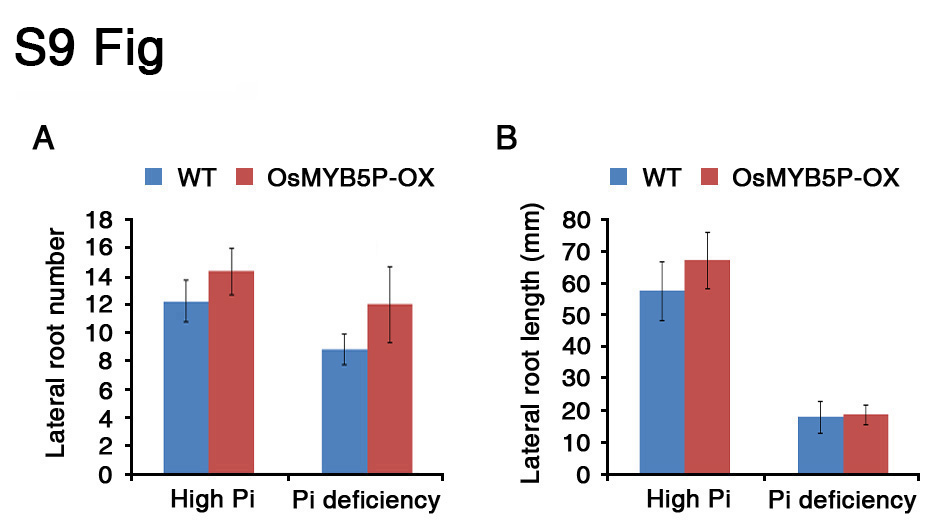

Supplement: S9 Fig — Total lateral root number (A) or length (B) on primary root of plants shown in Fig 5C. Error bars represent the mean ± SD of n = 6 replicates with 18 seedlings for each experiment. Asterisks represent significant differences from the WT (*; 0.01< p-value ≤ 0.05, **; p-value ≤ 0.01, Student’s t-test). (TIF) [file pone.0194628.s009.tif]

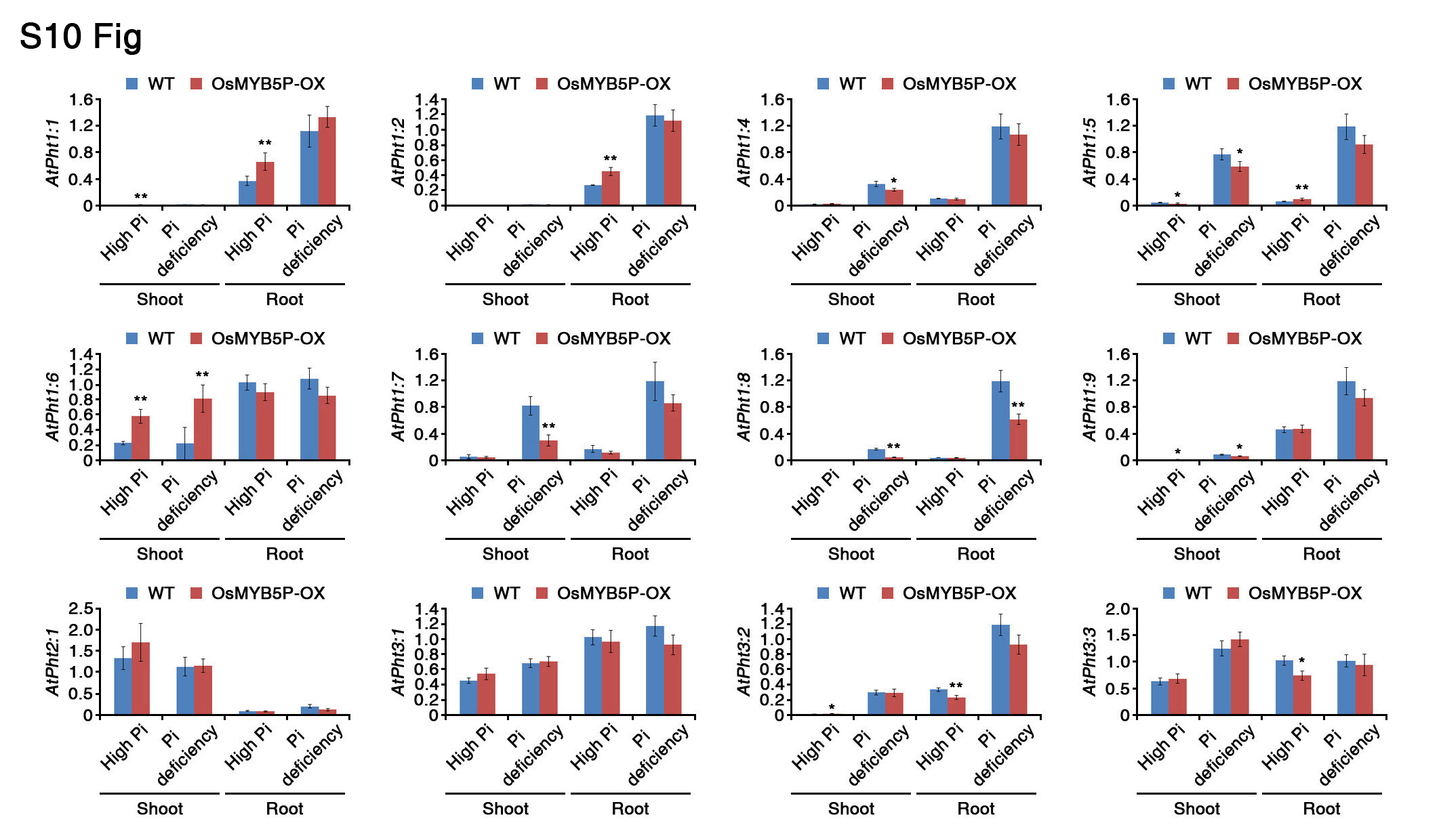

Supplement: S10 Fig — Total RNA was extracted from shoots and roots of Arabidopsis WT and OsMYB5P-OX seedlings grown under high Pi or Pi deficient conditions. Expression levels of AtTUBULIN2 were used for normalization. Bars represent the mean ± standard error of three technical replicates. Asterisks represent significant differences from the WT (*; 0.01 < p-value ≤ 0.05, **; p-value < 0.01, Student’s t-test). (TIF) [file pone.0194628.s010.tif]

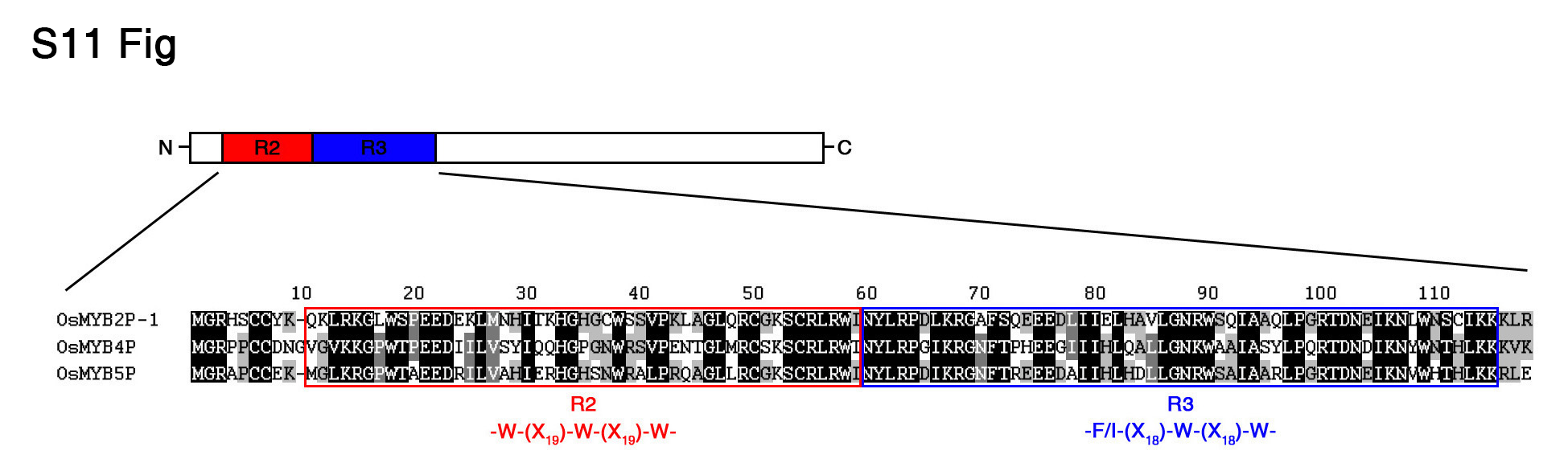

Supplement: S11 Fig — The two types of MYB domain repeats on the N terminus of OsMYB5P are indicated with red (R2) and blue (R3). The alignment of the DNA-binding (R2 and R3) domains with the amino acid sequence of rice R2R3-MYB transcription factors involved in Pi starvation responses was performed using CLUSTAL W. Identical amino acids are shaded black, and similar amino acids are shaded in gray. (TIF) [file pone.0194628.s011.tif]
