# Supplementary material for: Rice OsMYB5P improves plant phosphate acquisition by regulation of phosphate transporter
Source: PLoS One. 2018 Mar 22;13(3):e0194628. doi: 10.1371/journal.pone.0194628 (PMC5864048; doi:10.1371/journal.pone.0194628)
Supplement: S1 Table — (DOCX) [file pone.0194628.s012.docx]

**SUPPLEMENTARY MATERIALS_TABLES**

**S1 Table.** Putative MBS *cis*-elements in the promoter of rice phosphate transporters (*OsPTs*)

| Gene No. | Name | Position | Sequence | Strand |
| --- | --- | --- | --- | --- |
| Os3g0150600 | OsPT1 | NA^*^ | NA | NA |
| Os3g0150800 | OsPT2 | NA | NA | NA |
| Os10g0444600 | OsPT3 | -607 | TAACTG | - |
|  |  | -741 | TAACTG | - |
| Os4g0186400 | OsPT4 | -52 | TAACTG | - |
| Os4g0185600 | OsPT5 | -725 | CAACTG | - |
|  |  | -1098 | CAACTG | - |
| Os8g0564000 | OsPT6 | -682 | CGGTCA | - |
| Os3g0136400 | OsPT7 | -28 | CGGTCA | - |
| Os10g0444700 | OsPT8 | -457 | TAACTG | + |
|  |  | -992 | CAACTG | - |
|  |  | -1228 | CAACTG | + |
| Os6g0324800 | OsPT9 | -226 | CAACTG | - |
|  |  | -820 | TAACTG | + |
| Os6g0325200 | OsPT10 | No | NA | NA |
| Os1g0657100 | OsPT11 | -276 | CAACTG | + |
|  |  | -596 | CAACTG | + |
|  |  | -607 | CAACTG | - |
| Os3g0150500 | OsPT12 | -724 | TAACTG | - |

A gene region approximately 1,500 bp upstream of the transcriptional start site was analyzed using PlantCARE.

^*^ NA; Not Applicable.
