# Supplementary material for: Rice OsMYB5P improves plant phosphate acquisition by regulation of phosphate transporter
Source: PLoS One. 2018 Mar 22;13(3):e0194628. doi: 10.1371/journal.pone.0194628 (PMC5864048; doi:10.1371/journal.pone.0194628)
Supplement: S2 Table — (DOCX) [file pone.0194628.s013.docx]

**SUPPLEMENTARY MATERIALS_TABLES**

**S2 Table.** Putative MBS *cis*-elements in the promoter of *Arabidopsis* phosphate transporters (*AtPhts*)

| Gene No. | Name | Position | Sequence | Strand |
| --- | --- | --- | --- | --- |
| At5g43350 | AtPht1;1 | -958 | TAACTG | + |
| At5g43370 | AtPht1;2 | NA^*^ | NA | NA |
| At5g43360 | AtPht1;3 | -637 | TAACTG | + |
| At2g38940 | AtPht1;4 | -1299 | CAACTG | - |
|  |  | -710 | TAACTG | - |
|  |  | -646 | TAACTG | + |
| At2g32830 | AtPht1;5 | -136 | TAACTG | + |
| At5g43340 | AtPht1;6 | NA | NA | NA |
| At3g54700 | AtPht1;7 | NA | NA | NA |
| At1g20860 | AtPht1;8 | -1269 | TAACTG | - |
|  |  | -692 | CAACTG | - |
| At1g76430 | AtPht1;9 | NA | NA | NA |
| At3g26570 | AtPht2;1 | -1132 | TAACTG | - |
| At5g14040 | AtPht3;1 | NA | NA | NA |
| At3g48850 | AtPht3;2 | -881 | CAACTG | + |
| At2g17270 | AtPht3;3 | NA | NA | NA |

A gene region approximately 1,500 bp upstream of the transcriptional start site was analyzed using PlantCARE.

^*^ NA; Not Applicable.
