# Supplementary material for: Rice OsMYB5P improves plant phosphate acquisition by regulation of phosphate transporter
Source: PLoS One. 2018 Mar 22;13(3):e0194628. doi: 10.1371/journal.pone.0194628 (PMC5864048; doi:10.1371/journal.pone.0194628)
Supplement: S3 Table — (DOCX) [file pone.0194628.s014.docx]

**SUPPLEMENTARY MATERIALS_TABLES**

**S3 Table.** Primer lists for our studies

| Name | Forward primer (5'to3') | Reverse primer (5'to3') |
| --- | --- | --- |
| OsMYB5P-RNAi | CACCCGTCGTCCGGCCGGGAAGCC | CCGAGGTGAAACTGTCCGTG |
| OSMYB5P-OX | CACCATGGGGAGGGCGCCGTGCTG | TTATGCAGAATTTGCCCCAG |
| OsMYB5P-GST | CTCGAGAATGGGGAGGGCGCCGTG | CTCGAGTGCAGAATTTGCCCCAG |
| OsSQD-qRT | CTGAAAACGGTAATGGATAGG | AACAACAACAGCACGAGC |
| OsPAP10-qRT | ATACTGGCAGCCGACGGATGA | GAGGGAGCTGGAGCGGAGAA |
| OsmiR399j-qRT | GGAGCATGTAAGTCTTTTGTAGC | GGCAACTCTCCTTTGGCAGA |
| OsIPS-qRT | CTAAGGTAGGGCAACTTGTATC | TTATTAGAGCAAGGACCGAAAC |
| OsMYB5P-qRT | GTGGATCAACTACCTCCGC | TTCTTGATCTCGTTGTCCGTC |
| OsActin1-qRT | GAACTGGTATGGTCAAGGCTG | ACACGGAGCTCGTTGTAGAAG |
| OsPT5-qRT | TGCTACTGCCCATGACTAGGATT | CCATAGAAGAGATCCAGAGAAGCTGTA |
| OsPT3-MBS1-ChIP | GCTCCAAGCTCATTAACATAATCGTCTAACC | GGTCGGGTATATCCATATATGCGTTG |
| OsPT3-MBS2-ChIP | TTGACTAGGATTAAACAACATCCAAATGG | CGATTATGTTAATGAGCTTGGAGCTAAGG |
| OsPT4-MBS-ChIP | CTCAGTTCGACACATAGACATCTGAGG | GGCGCCAATCTCAGCTACTCTG |
| OsPT5-MBS1-ChIP | GTGCCTCCTATAGTAATAGAGAATGAAAACACC | AGAGAACATATGTTGGGTGCACAAAC |
| OsPT5-MBS2-ChIP | TAGAACTCATACCCACAAACTTAAACATTT | ATAGAGATTAAGACGAAGGAAAATCAAAAC |
| OsPT6-MBS1-ChIP | TGTCCGACCATCCCTTGCG | TCTCGCGAACGCGCTAAATG |
| OsPT6-MBS2-ChIP | TCGTAGTTGATTGATCAATCATCAATCTGT | AGGCTGGACAAGCCTTTCCTATCC |
| OsPT7-MBS-ChIP | ACACCGAACGCTACTACGATGTTGAT | ATCCAGCGGAAGCGCACC |
| OsPT8-MBS1-ChIP | ATGCAAGTTGAAATACAACTTCTACAAGTTAT | CAATCAGTTCACCCTAAAAGCTTTTAGTATA |
| OsPT8-MBS2-ChIP | ACTGTGGGAACCAAATTTCAGTGATG | TCTGGCTTGTGATTGGACCGTT |
| OsPT8-MBS3-ChIP | TAGATAGTACTATCTCCTAACCAACACCTCATGA | AGATATGATACTCTTATGTGAACGCAGATACAAT |
| OsPT9-MBS1-ChIP | AGTCGAAAGTCGATCGCATACGAA | AGCTTGCTCACTGGTGACGGC |
| OsPT9-MBS2-ChIP | TCACTAATTGCCAGTTTGCCACG | TACGAGCACCCATGCTAAATCTATACCA |
| OsPT11-MBS1-ChIP | ACGACGCATTTCCGTCCTAGTTAAC | GTACACTAGGCGTCTAGGCGGCA |
| OsPT11-MBS2-ChIP | ACAACTACCTTCAGCTTCTATTAGAGTTGGAG | TGGTAGGATATTCGGCAGATAATCATG |
| OsPT11-MBS3-ChIP | TACCGAATATGCCACTGAACTACCCA | ACCCTGCCGATCGATGCTCA |
| OsPT12-MBS-ChIP | AGCTGTTGTGCTCTAGCACATGTGC | GCTTGTGGTGACAGTGGAACCTATG |
| AtPht1;1-qRT | CTCTCAACGCCTCCTCAAGTTGAC | TGTAACGGGCAGTTTCAGGC |
| AtPht1;2-qRT | AAGGTGGATGCAGGATACCCA | GAACACCAAGCACGATCAATGA |
| AtPht1;3-qRT | GCTCAGTTGCTTCCGGTCTTT | ACCCGAGCCAAAACCTGAA |
| AtPht1;4-qRT | ACCCAATGCTACAACCTTCG | CTGGGTTCTGAGCCAAGTAC |
| AtPht1;5-qRT | GCCGCAAGAAAGTTTACGGTAT | GATAGACCAGACCCGAGAGAACA |
| AtPht1;6-qRT | CGGACTCCACTTACTCGGAACA | GCTGTAGAAAGCGATGTCGAGG |
| AtPht1;7-qRT | ATGTTTCTTCCGGTTCTGGCTT | CGTGGCGGATAACGGATAATC |
| AtPht1;8-qRT | ACTGCAGAAAACGTCTACGACG | CAGCGATGATGGCTCCTAATTC |
| AtPht1;9-qRT | CGTCGGTGAAAAGTCCCATTC | CGCAGCGAGGATACAGTGGTA |
| AtPht2;1-qRT | GCAGCTGGAACTTGGTTACAGG | CCAACCATTGATCCGACGATAC |
| AtPht3;1-qRT | TCGTTTCTCATCCAGCAGACAA | AATCTTCTTCACCGCATCTCCA |
| AtPht3;2-qRT | TTTAGCTGGATTGCCAACCACT | TACAGATGGAGCAAGCGCAGT |
| AtPht3;3-qRT | ATGTGTTGCAGGCTGTGAGGA | TGATCCGAACAGGAAGGCTTC |
| AtTubulin2-qRT | TGGCATCAACTTTCATTGGA | ATGTTGCTCTCCGCTTCTGT |
